# Implementation of the synthetic arabinose 5-phosphate dependent glycolaldehyde assimilation (SAGA) pathway in *Pseudomonas putida*

**DOI:** 10.3389/fbioe.2026.1875703

**Published:** 2026-08-25

**Authors:** Alrik Titze, Nils Wagner, Cláudio J. R. Frazão, Thomas Walther

**Affiliations:** Chair of Bioprocess Engineering, Institute of Natural Materials Technology, TU Dresden, Dresden, Germany

**Keywords:** acetyl-CoA, ethylene glycol assimilation, metabolic engineering, *Pseudomonas putida*, synthetic metabolic pathway

## Abstract

Ethylene glycol (EG) is a promising substrate for biochemical product syntheses as it can be sourced sustainably from CO_2_ or plastic waste. The synthetic, arabinose 5-phosphate dependent glycolaldehyde assimilation (SAGA) pathway has been recently developed to improve carbon efficiency for the conversion of EG to acetyl-CoA. This pathway performs poorly in *Escherichia coli,* because the initial NAD-dependent EG oxidation is thermodynamically unfavorable (ΔrG′° = +23.7 kJ mol^−1^), resulting in slow and incomplete consumption of the substrate. Transferring the pathway to *Pseudomonas putida* KT2440 overcomes this barrier, as its native PQQ-dependent dehydrogenases PedE and PedH oxidize EG irreversibly. Here, the SAGA pathway was extended to enable the formation of mevalonate (MVA). Carbon tracing experiments were used to quantify the contribution of glucose metabolism and synthetic or native EG-assimilating pathways to the formation of acetyl-CoA. Deletion of the two known GA dehydrogenases (*aldB-I* and *aldB-II*) resulted in detectable SAGA pathway activity which accounted for 2.5% of acetyl-CoA production but did not prevent its formation through EG assimilation *via* the native pathway (10.0%). To improve GA assimilation, we identified all GA reductases in KT2440, which when deleted, eliminated GA to EG conversion but did not further improve the performance of the SAGA pathway. Limited regeneration of the GA-acceptor glyceraldehyde-3-phosphate (GA3P) was addressed by supplying glucose as a co-substrate. Deletion of the PQQ-dependent glucose dehydrogenase *gcd* alleviated competition of Gcd and PedE for the cofactor, permitting simultaneous uptake of EG and glucose. Optimized expression of all SAGA pathway genes raised the contribution of the synthetic pathway to 3.0% of total acetyl-CoA production. Additional removal of the major GA3P dehydrogenase *gapA* increased the SAGA pathway’s contribution by 91% to 5.7%. When glucose was provided at reduced rates using feed beads, the share of EG-derived acetyl-CoA increased to 17.3% out of which 9.3% were channeled through the SAGA pathway. However, under these conditions the total MVA accumulation after 24 h dropped from 2.9 mM observed for the high-glucose condition to 0.3 mM.

## 1 Introduction

Microbial fermentation plays a critical role in sustainable industrial chemical production within a circular economy, which aims at reducing the reliance on fossil resources (Clomburg et al., 2017). This requires the utilization of carbon feedstocks which do not compete with food production unlike the commonly utilized energy-plant derived sugars (Gustavsson and Lee, 2016). Amid efforts to reduce greenhouse gas emissions, CO_2_ stands as an underutilized source of carbon for microbial production of chemicals (Gavala et al., 2021). One approach to facilitate its utilization is the chemical conversion of CO_2_ to more reduced, fermentable one-carbon (C_1_) substrates, most prominently methanol or syngas (Goeppert et al., 2014; Cotton et al., 2020; Zhan et al., 2021). However, microbial production processes using C_1_ substrates encounter challenges including restricted range of products (Takors et al., 2018; Teixeira et al., 2018), poor mass transfer rates and low water solubility of gaseous substrates (Frazão and Walther, 2020), whereas methanol-based processes tend to have a high oxygen demand. In the search for alternative, CO_2_-derived platform chemicals for microbial product syntheses, ethylene glycol (EG) receives growing interest (Wagner et al., 2023b; Shimizu and Inui, 2024). The C_2_ alcohol can be sustainably sourced through various strategies including the electro-chemical conversion of CO_2_ *via* ethylene (Prajapati et al., 2022; Fan et al., 2023), ethylene oxide (Li et al., 2022) or syngas (Sun and Chai, 2022; Gor et al., 2023), but also through the enzymatic digestion of PET waste (Ellis et al., 2021). EG-based biosynthesis avoids a lot of the aforementioned challenges due to its high solubility in water (Yue et al., 2012), a lower oxygen demand compared to methanol-based processes, and the possibility to produce a variety of products without thermodynamic restrictions (Wagner et al., 2023b).

**FIGURE 1:**
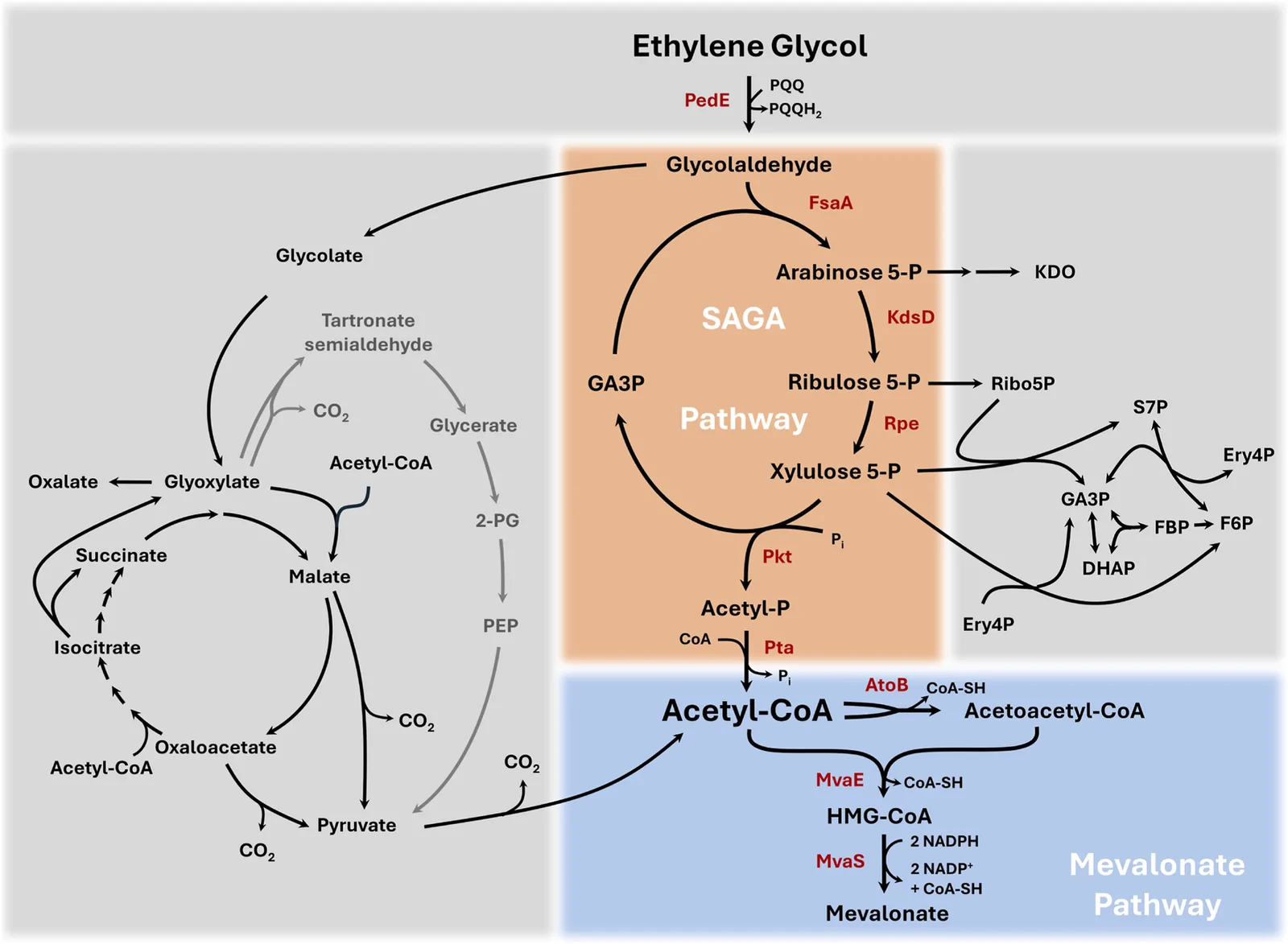
Synthetic, arabinose 5-phosphate dependent glycolaldehyde assimilation (SAGA) pathway and mevalonate (MVA) pathway in cellular context of *Pseudomonas putida* KT2440. Naturally occurring reactions are depicted on a gray, SAGA pathway reactions on a red and MVA pathway reactions on a blue background. The glycolate pathway from glyoxylate to pyruvate is shown in gray, as it is inactive in the KT2440 strain. Genes required for SAGA and MVA pathways are shown in red and co-factors are only shown for SAGA and MVA pathway reactions. Abbreviations: 2-PG, 2-Phosphoglycerate; Acetyl-CoA, Acetyl-Coenzyme A; Acetyl-P, Acetyl phosphate; Arabinose 5-P, arabinose 5-phosphate; DHAP, dihydroxyacetone phosphate; Ery4P, erythrose 4-phosphate; F6P, fructose 6-phosphate; FBP, fructose 1,6-bisphosphate; GA3P, glyceraldehyde 3-phosphate; HMG-CoA, 3-hydroxy-3-methylglutaryl coenzyme A; KDO, 3-deoxy-α-D-manno-2-octulosonate; PEP, phosphoenolpyruvate; Ribo5P; ribose 5-phosphate; Ribulose 5-P, ribulose 5-phosphate; S7P, sedoheptulose 7-phosphate; Xylulose 5-P, Xylulose 5-phosphate.

In various microbial species, EG is naturally assimilated *via* a sequence of oxidation reactions proceeding through the intermediates glycolaldehyde (GA), glycolate and glyoxylate which then enters the central carbon metabolism *via* the glyoxylate shunt or conversion to 2-phosphoglycerate (Mückschel et al., 2012; Franden et al., 2018; Shimizu and Inui, 2024). The latter is produced through the intermediates tartronate semialdehyde and D-glycerate (Figure 1). The formation of tartronate semialdehyde requires the decarboxylation of one glyoxylate molecule, which is fused to a second glyoxylate molecule yielding the C_3_ intermediate and CO_2_. Alternatively, malate is generated by adding glyoxylate to acetyl-CoA through the glyoxylate shunt. The regeneration of acetyl-CoA yields 2 mol of NAD(P)H but this pathway does not allow for biomass or product formation, as it contains two subsequent decarboxylation reactions which emit the carbon initially provided by the C_2_ compound EG in the form of CO_2_. These high carbon losses in the native pathways are the key motivation for the development of synthetic EG-based production pathways with increased carbon-efficiencies (Frazão et al., 2023; Wagner et al., 2023b; Frazão et al., 2025).

In this context, a carbon-conserving reaction sequence for the conversion of EG into acetyl-CoA, first described by Yang et al., 2019, is of particular interest, since this metabolic hub molecule serves as a precursor for a wide range of value-added products (Krivoruchko et al., 2015; Liao et al., 2016). The so-called synthetic arabinose 5-phosphate-dependent GA assimilation (SAGA) pathway starts with the formation of D-arabinose 5-phosphate (Ara5P) through cross-aldol addition of GA to glyceraldehyde 3-phosphate (GA3P). Two successive reactions catalyzed by Ara5P isomerase (KdsD) and D-ribulose 5-phosphate epimerase (Rpe) then rearrange the phosphorylated sugar into D-xylulose 5-phosphate (Xylu5P). Xylu5P is cleaved in a phosphoketolase (Pkt) catalyzed reaction, regenerating the GA acceptor GA3P and producing acetyl phosphate, which is finally converted to acetyl-CoA by a phosphate acetyltransferase (Pta) (Figure 1). The LC/MS-method in our lab did not facilitate the reliable quantification of acetyl-CoA formation. Therefore, we extended the SAGA pathway by a mevalonate (MVA) pathway comprising enzymatic activities of acetyl-CoA acetyltransferase, acetoacetyl-coenzyme A thiolase and MVA synthase. MVA is a stable compound which is exclusively derived from acetyl-CoA, thus, the contribution of the SAGA pathway to the formation of acetyl-CoA could be inferred from the labeling pattern of MVA in ^13^C-based carbon-tracing experiments. The feasibility of both the SAGA and the MVA pathway has been shown *in-vitro* (Xiong et al., 2014; Yang et al., 2019; Wagner et al., 2023a) and *in-vivo* using an engineered *Escherichia coli* strain (Wagner et al., 2023a). These experiments showed poor pathway performance when EG was used as a starting substrate. In large part, this was due to the thermodynamically unfavorable NAD-dependent oxidation of EG to GA, which has a highly positive standard Gibbs energy (Δ_r_G’° = 23.7 kJ mol^−1^) preventing efficient EG assimilation. In contrast, *P. putida* KT2440 possesses two PQQ-dependent alcohol dehydrogenases (PedE and PedH), which naturally oxidize EG. This PQQ-dependent reaction has a highly negative Gibbs energy (Δ_r_G’° = −42.4 kJ mol^−1^) and may therefore improve the performance of the SAGA pathway (Mückschel et al., 2012; Wehrmann et al., 2017). Furthermore, *Pseudomonas putida* has already been engineered to convert EG into biomass (Li et al., 2019) but also value-added products such as medium chain length polyhydroxyalkanoates (Franden et al., 2018), underlining the potential of this strain to host EG-dependent pathways.

In this study, we investigated the SAGA pathway’s *in-vivo* performance by exploiting the thermodynamically favorable PQQ-dependent EG oxidation in *P. putida*. Supplementation of the culture media with the co-substrate glucose was required to regenerate the GA-acceptor molecule GA3P. Simultaneous assimilation of EG and the co-substrate glucose was enabled by alleviating the competition for the PQQ-cofactor through deletion of the PQQ-dependent glucose dehydrogenase (Gcd). Upon deletion of two competing native GA-dehydrogenases, and downregulation of natural GA3P conversion, the majority of EG derived flux was redirected from native EG-assimilation reactions to the engineered SAGA pathway. However, since the SAGA pathway contribution was limited to 9.3% of total acetyl-CoA, the presented findings serve as a proof-of-concept which highlight opportunities for further pathway optimization.

## 2 Materials and methods

### 2.1 Reagents and chemicals

Chemicals and solvents were purchased from Sigma-Aldrich (St. Louis, MO, United States) unless otherwise stated. [1,2–^13^C_2_] Ethylene glycol (isotopic enrichment 99%) was purchased from Eurisotop (Saint-Aubin, France). DNA plasmid isolation and DNA extraction from agarose gels was performed using the Monarch® Kit from New England Biolabs (Ipswich, MA, United States). DNA Sanger sequencing was carried out by Genewiz (Leipzig, Germany) or Eurofins SAS (Ebersberg, Germany).

### 2.2 Strains and plasmids

All strains used in this study are listed in Table 1. Plasmids used for genomic deletions, gene integrations and gene expression are listed in Supplementary Table S1.

**TABLE 1:** Strains used in this study.

Strain	Genotype	Source
BL21 (DE3)	*E. coli fhuA2 [lon] ompT gal (λ DE3) [dcm] ΔhsdS* *λ DE3 = λ sBamHIo ΔEcoRI-B int::(lacI::PlacUV5::T7 gene1) i21 Δnin5*	NEB™
NEB5-α	*E.coli fhuA2Δ(argF-lacZ)U169 phoA glnV44 Φ80Δ(lacZ)M15 gyrA96 recA1 relA1 endA1 thi-1 hsdR17*	NEB™
NEB stable	*E.coli F′ proA* ^*+*^ *B* ^*+*^ * lacI* ^*q*^ * Δ(lacZ)M15 zzf::Tn10 (Tet* ^*R*^ *)/Δ(ara-leu) 7697 araD139 fhuA ΔlacX74 galK16 galE15 e14- Φ80dlacZΔM15 recA1 relA1 endA1 nupG rpsL (Str* ^*R*^ *) rph spoT1 Δ(mrr-hsdRMS-mcrBC)*	NEB™
SM10(λpir)	*E.coli thi thr leu tonA lacY supE recA::RP4-2-Tc::Mu Km λpir*	BCCM, Belgium
WM3064	*E. coli thrB1004 pro thi rpsL hsdS lacZΔM15 RP4-1,360 Δ(araBAD)567 ΔdapA1341::[erm pir]*	(Lassak et al., 2010)
*P. putida* KT2440 wildtype	*P. putida* KT2440	DSMZ GmbH, Germany
*P .putida* KT2440 *ΔpedE::syfp2*	*P .putida* KT2440 *ΔpedE::syfp2*	This work
SMP1	*P. putida* KT2440 PP1035::J23100-Ec.atoB-Lc.mvaS-Lc.mvaE PP5322::J23100-ec.pta + pBTL2_ec.fsaA(c-opt)-ca.pkt(c-opt)	This work
SMP2	SMP1 *ΔaldB-I ΔaldB-II*	This work
SMP3	*S*MP2 ΔPP_2426 ΔPP_3839 ΔPP_2492	This work
SMP4	*SMP2 Δgcd* PP1035::J23100-Ec.atoB-Lc.mvaS-Lc.mvaE PP5322::J23100-ec.pta + pBTL2_ec.fsaA(c-opt)-ca.pkt(c-opt)	This work
SMP5	*SMP2 Δgcd* PP1035::J23100-Ec.atoB-Lc.mvaS-Lc.mvaE PP5322::J23100-ec.pta PP5042::J23100-ec.kdsD(c-opt)-ec.rpe + pBTL2_ec.fsaA(c-opt)	This work
SMP6	*SMP2 Δgcd* PP1035::J23100-Ec.atoB-Lc.mvaS-Lc.mvaE PP5322::J23100-ec.pta PP5042::J23100-ec.kdsD(c-opt)-ec.rpe + pBTL2_ca.pkt(c-opt)	This work
SMP7	*SMP2 Δgcd* PP1035::J23100-Ec.atoB-Lc.mvaS-Lc.mvaE PP5322::J23100-ec.pta PP5042::J23100-ec.kdsD(c-opt)-ec.rpe + pBTL2_ec.fsaA(c-opt)-ca.pkt(c-opt)	This work
SMP8	SMP7 *ΔglcDEF*	This work
SMP9	SMP7 *ΔgapA*	This work
SMP10	*P. putida* KT2440 *ΔaldB-I ΔaldB-II Δgcd ΔgapA* PP1035::J23100-Ec.atoB-Lc.mvaS-Lc.mvaE PP5322::J23100-ec.pta PP5042::J23100-ec.kdsD(c-opt)-ec.rpe + pBTL2_ec.fsaA(c-opt)-ca.pkt(c-opt)- Ec.atoB-Lc.mvaS-Lc.mvaE	This work
Red0	*P. putida* KT2440 *ΔaldB-I ΔaldB-II*	This work
Red0 Δ…	Red0 carrying indicated deletions of glycolaldehyde dehydrogenase candidate genes	This work
Red1	Red0 ΔPP_2426 ΔPP_3839 ΔPP_2492	This work

### 2.3 Media

Liquid lysogeny broth (LB) medium (10 g L^−1^ tryptone, 5 g L^−1^ yeast extract, 10 g L^−1^ NaCl) served as the culture medium for cloning workflows, strain viability preservation and cell recovery from glycerol cultures (25% v/v) stored at −80 °C.

Unless otherwise stated, *in-vivo* experiments were carried out in M9 minimal medium prepared 18 g L^−1^ Na_2_HPO_4_ · 12 H_2_O, 3 g L^−1^ KH_2_PO_4_, 0.5 g L^−1^ NaCl, 2 g L^−1^ NH_4_Cl, 0.5 g L^−1^ MgSO_4_, 0.015 g L^−1^ CaCl_2_ · 2 H_2_O, 0.01 g L^−1^ FeCl_3_, 0.012 g L^−1^ Thiamine HCl, 0.4 g L^−1^ Na_2_EDTA · 2 H_2_O, 1.8 mg L^−1^ CoCl_2_ · 6H_2_O, 1.8 mg L^−1^ ZnSO_4_ · 7 H_2_O, 0.4 mg L^−1^ Na_2_MoO_4_ · 2 H_2_O, 0.1 mg L^−1^ H_3_BO_3_, 1.2 mg L^−1^ MnSO_4_ · H_2_O, 1.2 mg L^−1^ CuCl_2_ · 2 H_2_O according to Walther et al., 2017. To buffer the medium, 100 mM 3-(*N*-morpholino)-propanesulfonic acid (MOPS) adjusted to pH seven using KOH were added. Carbon sources were supplemented as described in the method description for shake flask cultivations.

Whenever required, kanamycin sulfate was added to liquid and solid media to a final concentration of 50 mg L^−1^.

### 2.4 Construction of pNPTS138-R6KT derived suicide vectors

Gene deletions and chromosomal integrations in *P. putida* KT2440 were carried out using pNPTS138-R6KT (Lassak et al., 2010) suicide vectors which are unable to replicate in *P. putida* KT2440 and contain both a kanamycin resistance gene, and a *sacB* gene inducing sucrose sensitivity for counter-selection. *Escherichia coli* DH5α-λpir (Lassak et al., 2010) was used for construction and storage of pNPTS138-R6KT plasmids. To facilitate allelic exchange, sequences homologous to 500–1,000 base pairs up and downstream of the deletion gene or integration target site were integrated into the plasmid’s multiple cloning site. To facilitate gene integration the integration cassette containing gene sequences, the constitutive promoter J23100 (Sanches-Medeiros et al., 2018; Amarelle et al., 2019), gene-specific RBS and flanking terminators (Chaves et al., 2020) were inserted between the homologous sequences. For a detailed description of plasmid construction, see the supplementary information. Briefly, the desired sequences were integrated into the suicide vector following PCR-restriction cloning or PCR amplification using primers containing 10–20 base pair tails for subsequent *in-vitro* DNA assembly using NEBuilder HiFi DNA Assembly kit (New England Biolabs).

### 2.5 Construction of pBTL2 derived plasmids for gene expression in *Pseudomonas putida* KT2440

For plasmid-based enzyme overexpression of Ara5P-SAGA pathway and MVA pathway genes in *P. putida* KT2440, broad host range pBTL2 (pBBR1 origin, Kan resistance, P_Lac_ promoter) (Lynch and Gill, 2006) expression plasmids were used. Plasmids were constructed and stored in NEB 5-alpha cells (New England Biolabs). Target genes were cloned into the multiple cloning site downstream of P_Lac_ promoter using NEBuilder Hifi DNA Assembly kit (New England Biolabs). For a more detailed explanation of plasmid construction see the supplementary information. Briefly *Ec. fsaA* and *Ca. pkt* were PCR amplified from codon optimized gene sequences, MVA pathway genes were amplified from the pMEV-7 vector (Xiong et al., 2014). Primers contained 10–20 bp tails for subsequent *in-vitro* DNA assembly.

### 2.6 Strain construction

Deletion and chromosomal integration of genes in *P. putida* KT2440 were accomplished following the protocol of Lassak et al., 2010. To this end, pNPTS138-R6KT derived suicide vectors were introduced into *P. putida* KT2440 strains by conjugative mating from diaminopimelic acid (DAP) auxotrophic *E. coli* WM3064 (Dehio and Meyer, 1997). Briefly, cell mass from *P. putida* KT2440 deletion target strains and from *E. coli* WM3064 carrying the suicide vectors grown on solid medium were resuspended in 100 µL phosphate buffered saline (PBS; 0.16 g L^−1^ NaH_2_PO_4_ · H_2_O; 0.2 g L^−1^ Na_2_HPO_4_ · 12 H_2_O; 8.1 g L^−1^ NaCl; adjusted to pH 7). Both resuspensions were mixed at a ratio of 1:3 vector donor to vector recipient strain and 20 µL of the mixture were spotted on LB agar plates containing 0.25 g L^−1^ DAP. After incubation at 30 °C for 8 h, the resulting cell mass was resuspended in 100 µL PBS and plated on LB agar plates supplemented with kanamycin to select for *P. putida* KT2440 integration mutants. Single colonies were confirmed to be sensitive to 100 g L^−1^ sucrose and grown in LB medium for 6–8 h at 30 °C. Then, 100 µL of a 1:10 dilution was plated on LB agar plates supplemented with 100 g L^−1^ sucrose to select for plasmid excision as the result of a second crossover event, and incubated at room temperature for 1–2 days. Single colonies were transferred to LB agar plates and screened for kanamycin sensitivity. Finally, kanamycin sensitive colonies were analyzed for deletion of the target locus or successful integration by colony PCR using DreamTaq® DNA polymerase. Chromosomal integrations were additionally verified by Sanger sequencing.

### 2.7 Preparation of cell-free extracts and protein quantification

Cell-free extracts were prepared from *P. putida* KT2440 strains which were first grown in LB medium at 30 °C and 220 rpm for 8 h. Then 25 mL of M9 medium supplemented with 111 mM glucose were inoculated with 1 mL of LB pre-culture and grown overnight at 30 °C and 220 rpm. The next day, 50 mL of M9 medium supplemented with 111 mM glucose and kanamycin were inoculated to an OD_600_ of 0.2 and cultivated at 30 °C and 220 rpm. When an OD_600_ of 1.8–2 was reached, cells were harvested by centrifugation at 8,000 g and 4 °C for 10 min. Pellets were stored at −20 °C. To prepare cell-free extracts, pellets were thawed and resuspended in 500 µL of an assay specific buffer (F6P aldolase (FsaA) assay: 25 mM HEPES, pH 7; Phosphoketolase (Pkt) assay: 13.3 mM HEPES + 6.7 mM KH_2_PO_4_, pH 7; Phosphate acetyltransferase (Pta) assay: 5 mM Tris-HCl, pH 8). Cells were then sonicated in three successive 10 s sonication intervals at 30% power output (Topas, UDS 751) and 30 s of pause. The extracted supernatant was separated from cellular debris by centrifugation at 13,000 g and 4 °C for 5 min. The concentration of proteins in this crude extract was determined by Bradford assay (ROTI®Quant, Roth) using bovine serum albumin to generate a standard calibration curve (0–100 μg mL^−1^).

### 2.8 Measurement of *in-vitro* enzyme activities

The functional expression of enzymes was confirmed by measuring activities on corresponding natural substrates as described in the supplementary information.

### 2.9 Shake flask cultivation of *Pseudomonas putida* KT2440 strains

*Pseudomonas putida* KT2440 cells were cultivated in orbital shakers (Ecotron, Infors) at a shaking frequency of 220 rpm and 30 °C. Liquid and solid media were supplemented with kanamycin when required. Frozen glycerol stocks were streaked on LB agar plates. These plates were then used to inoculate a first pre-culture of 5 mL liquid LB medium and cultivated for 8 h. Cells from the first pre-culture were then diluted 25-fold in 50 mL M9 medium supplemented with 111 mM glucose and grown in a second pre-culture overnight to adapt to the minimal medium. In high-cell-density experiments with slow growing strains, LB and M9 pre-culture durations were extended to reach OD_600_ > 2 before initiating the main culture.

For MVA production cultivations, three different main culture medium compositions were used, differing in the availability of co-substrate glucose: (1) Medium without co-substrate, where no glucose was supplemented; (2) medium where 2 glucose-releasing beads (12 mm polymer disc, Adolf Kuhner AG, Basel, Switzerland) were added, which allow for slow, controlled release of glucose (25 mM after 48 h, at a rate of ≈ 0.5 mM h^−1^); and (3) medium where glucose was supplemented at a concentration of 111 mM. The main cultures were either inoculated at low-cell-density or high-cell-density. For low-cell-density inoculation, medium from the second pre-culture was used to inoculate the main culture to a starting OD_600_ of 0.2. For high-cell-density inoculation, cells were harvested by centrifugation at room temperature and 4,500 g for 10 min. Pellets were resuspended in 1–2 mL of deionized water to allow for main culture inoculation at starting OD_600_ of 10–14.

To identify GA reductases, cells from the second pre-culture were harvested by centrifugation (4,500 g for 10 min at 20 °C). Main cultures were inoculated to an OD_600_ of 0.2 and cultivated in 10 mL M9 medium supplemented with 111 mM glucose. During exponential growth (OD_600_ ∼ 1.2), 10 mM GA was added to the medium.

To grow cell mass for the analysis of PedE transcription in the presence of glucose *via* fluorescence measurements, first and second pre-culture were prepared as previously described. For the main-culture, 10 mL M9 medium supplemented with 33.33 mM glucose and either 320 mM EG or no EG were inoculated with second pre-culture to an initial OD_600_ of 0.5.

### 2.10 Fluorescence measurements to quantify *pedE* activation

Samples extracted from shake flasks were diluted with deionized water to a fixed OD_600_ of 0.5 and 100 µL were transferred to black, clear-bottom 96-well plates (Corning Inc., NY, United States). Fluorescence was measured using a microplate reader (GENios™, Tecan, Switzerland) at an excitation wavelength of 485 nm and a detection wavelength of 535 nm (Gain: 80, 10 flashes per well, 2 mm shaking amplitude). Fluorescence intensity of *P. putida ΔpedE*::syfp2 was subtracted from the values determined for *P. putida* wildtype cultivated in parallel, in the same conditions.

### 2.11 Quenching and extraction of intracellular metabolites

At appropriate time intervals, 0.5 mL of cell culture medium were withdrawn and vacuum filtered (polyamide filter, 0.2 µm pore size, Sartorius Stedim) and washed by the addition of 1 mL room temperature wash solution consisting of deionized water and 111 mM glucose. Filters with cells were then immediately transferred to glass tubes containing 5 mL 75% (v/v) ethanol pre-heated to 80 °C and incubated for 3 min. Then, filter-containing tubes were transferred on ice and chilled for another 10 min. The filter was removed, the ethanol was subdivided into 2 mL microtubes and centrifuged (13,000 g, room temperature) for 5 min to remove cell debris and filter remnants. The supernatant was stored overnight at −20 °C. The next day, liquid was evaporated at 45 °C for 4 h using a benchtop vacuum concentrator (CentriVap Concentrator System, Labonco, United States). Dried metabolites were resuspended in 250 µL deionized water and analyzed directly by LC/MS.

### 2.12 Quantification of extracellular and intracellular metabolites

Extracellular metabolites were quantified by high performance liquid chromatography (UltiMate 3000 system, Dionex, United States). The installed autosampler (Dionex, United States) cooled samples to 4 °C. Components were detected *via* a coupled UV/Vis detector (Dionex, United States) measuring at 210 nm and an RI detector (ERC RefractoMax 520, Knauer, Germany). ^13^C labeling was quantified by coupling liquid chromatography (Vanquish™, ThermoFisher™, United States) with a mass spectrometer (Q Exactive™ Focus orbitrap, ThermoFisher™, United States).

For the analysis of extracellular metabolites, samples from shake flask cultivations were centrifuged (5 min at 13,000 rpm), syringe filtered (0.2 µm, PTFE, ThermoFisher, United States) and stored at −20 °C. Components of the cell free supernatant were separated using Rezex ROA-Organic Acid H+ column (300 × 7.8 mm, 8%, Phenomenex) and protected by a pre-column (Security Guard Carbo H^+^, 4 × 3 mm, Phenomenex). Analytes were eluted with 0.5 mM H_2_SO_4_, at a constant flow rate of 0.5 mL min^−1^. The column temperature was adjusted to 80 °C.

For the analysis of intracellular metabolites, samples from shake flask cultivations were quenched and processed as described above. All resuspended samples and standards were prepared in a matrix of 60% (v/v) acetonitrile and 40% (v/v) water mixed with ammonium acetate (pH 9.2) to a final concentration of 10 mM. Samples were separated using a SeQuant® ZIC®-pHILIC column (5 µm polymer 150 × 2.1 mm). To optimize separation efficiency, a gradient of Eluent A (5% acetonitrile, 10 mM ammonium acetate, pH 9.2 adjusted by NH_4_OH) and Eluent B (90% acetonitrile, 10 mM ammonium acetate, pH 9.2 adjusted by NH_4_OH) was used. The gradient was as follows: 0 min, 95% B; 2 min, 95% B; 3 min, 89.4% B; 5 min, 89.4% B; 6 min, 83.8% B; 7 min, 83.8% B; 8 min, 78.2% B; 9 min, 78.2% B; 10 min, 55.9% B; 12 min, 55.9% B; 13 min, 27.9% B; 16 min, 27.9% B; 18 min, 0% B; 23 min, 0% B; 24 min, 95% B; 30 min, 95% B adjusted to a flow rate of 0.15 mL min^−1^. Samples were cooled to 6 °C in the autosampler, injection volume was 5 μL and 25 °C was set as the oven temperature. Mass spectrometer instrumental settings were optimized for the specific flow rate as follows: sheath gas flow rate 32 (arbitrary units), auxiliary gas flow rate 8 (arbitrary units), sweep gas flow rate 0 (arbitrary units), spray voltage −3.5 kV, capillary temperature 250 °C and auxiliary gas temperature 200 °C. Metabolites were identified by their monoisotopic mass and retention time identified using unlabeled standards. Peak areas were corrected for the naturally occurring ^13^C isotopes using the software IsoCor (Millard et al., 2012).

#### 2.12.1 Relative ^13^C-enrichment in MVA

Relative ^13^C-enrichment in MVA is here defined as the fraction of detected labeled carbon relative to the total amount of carbon in MVA. It is calculated as the sum of relative abundances of each isotopologue fraction multiplied by their specific ratios of labeled carbon atoms to total carbon atoms.

#### 2.12.2 Inferred ^13^C labeling in acetyl-CoA

Assuming one MVA molecule is produced through the random combination of three acetyl-CoA molecules, a predicted MVA isotopologue distribution was calculated from an acetyl-CoA isotopologue distribution using Formulas 1–7. Isotopologues M+a of MVA are written as M_a_ and isotopologues M+b of acetyl-CoA are written as m_b_. The acetyl-CoA isotopologue distribution was then iteratively improved by minimizing the sum of squared differences between predicted MVA isotopologue distribution and measured MVA isotopologue distribution using a generalized reduced gradient algorithm.

$$M_{0}={m_{0}}^{3}$$ (1)

$$M_{1}={{3\;m}_{0}}^{2}m_{1}$$ (2)

$$M_{2}={{3\;m}_{0}}^{2}m_{2}+{{3m_{0}m}_{1}}^{2}$$ (3)

$$M_{3}={6\;m}_{0}m_{1}m_{2}+{m_{1}}^{3}$$ (4)

$$M_{4}={3\;m}_{0}{m_{2}}^{2}+{{3m}_{1}}^{2}m_{2}$$ (5)

$$M_{5}={3\;m}_{1}{m_{2}}^{2}$$ (6)

$$M_{6}={m_{2}}^{3}$$ (7)

## 3 Results and discussion

### 3.1 Construction and initial characterization of the base strain for operation of the SAGA pathway

The objective of this work was the *in-vivo* implementation, characterization and optimization of the SAGA pathway in *P. putida*. Since acetyl-CoA could not be reliably quantified by the analytical methods established in our lab, a pathway to produce the stable isoprenoid-precursor MVA from three acetyl-CoA molecules was additionally expressed in the producer strain. Quantifying the extracellular MVA concentration and analyzing the ^13^C labeling patterns resulting from experiments using uniformly labeled EG served to infer the contribution of the SAGA pathway to the total acetyl-CoA production.

For the conversion of GA and GA3P into Ara5P, we utilized fructose 6-phosphate aldolase (FsaA) from *E. coli* (Figure 1). In the initial *in-vitro* implementation of the SAGA pathway by Yang et al., 2019, a TalB^F178Y^ mutant was employed for this cross-aldol addition. However, *in-vivo* pathway performance in *E. coli* increased when FsaA was used instead (Wagner et al., 2023a). The formation of acetyl phosphate and regeneration of GA3P from Xylu5P was catalyzed by a phosphoketolase (Pkt) from *Clostridium acetobutylicum* due to its strong preference for Xylu5P over F6P (Bergman et al., 2016). Regarding the remaining reactions of the SAGA pathway, *P. putida* KT2440 natively possesses both enzymes with the required activities: Ara5P isomerase (KdsD, PP_0957) and D-ribulose 5-phosphate 3-epimerase (Rpe, PP_0415). While *P. putida* KT2440 also possesses a native phosphate acetyltransferase (Pta), its activity has been reported to be six times lower than the native Pta enzyme of *E. coli* (Nikel and De Lorenzo, 2013). Furthermore, *in-vivo* characterization of the SAGA pathway in *E. coli* revealed a bottleneck imposed by endogenous Pta activity, which was relieved by Ec. Pta overexpression (Wagner et al., 2023a). Because of these findings, we also overexpressed Ec. Pta in our producer strain. MVA formation was enabled by expressing the acetyl-CoA acetyltransferase from *E. coli* (Ec.AtoB) in addition to acetoacetyl-coenzyme A thiolase and MVA synthase from *Lactobacillus casei* (Lc.MvaE and Lc.MvaS). The feasibility of producing MVA using these genes has been demonstrated in *P. putida* (Zhang et al., 2024). To enable sufficient expression of these genes, the heterologous *Ec. fsaA* and *Ca. pkt* genes were expressed from a medium copy pBTL2 plasmid, while *Ec. pta* and the MVA pathway genes were integrated into the *P. putida* KT2440 chromosome to yield the initial production strain SMP1.

After verifying by enzymatic assays that all heterologous pathway genes were functionally expressed (Supplementary Figure S1), we set out to characterize the behavior of the base strain in shake flask experiments.

Since *P. putida* KT2440 was reported to be unable to grow on EG alone (Mückschel et al., 2012), the effect of supplementing the culture medium with the co-substrate glucose, either provided at high concentrations (111 mM) or continuously released from glucose feed beads (average feed-rate: 0.5 mM h^−1^) was investigated. Experiments were carried out by inoculating the cultivation medium at low cell densities (LCD, OD_600_ = 0.2) and high cell densities (HCD, OD_600_ ≈ 12), to test whether cells could grow and/or form product under these conditions.

**FIGURE 2:**
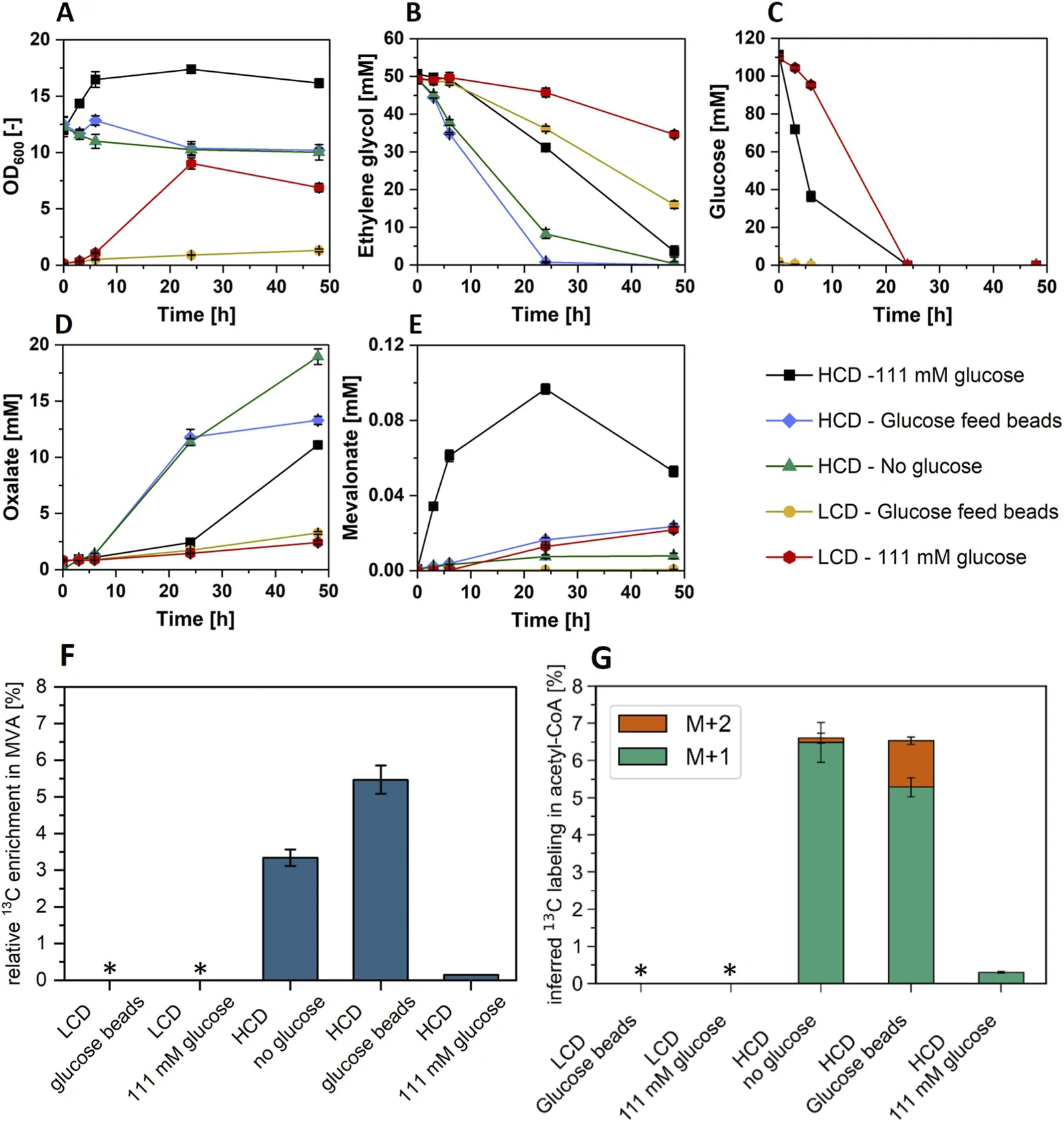
Growth and metabolite concentrations of SMP1 (*Pseudomonas putida* KT2440–Ec.atoB-Lc.mvaS-Lc.mvaE-Ec.KdsD(c-opt)-Ec.Rpe-Ec.Pta + pBTL2-Ec.FsaA(c-opt)-Ca.Pkt(c-opt)), depending on initial cell density and presence of glucose. Cells were inoculated at high cell density (HCD, OD_600_ ∼12) or low cell density (LCD, OD_600_ = 0.2) and cultivated in M9 + 50 mM ^13^C_2_-EG supplemented with either 111 mM glucose, glucose-releasing beads or no glucose at 30 °C. The LCD condition without glucose supplementation was omitted due to absence of growth and negligible substrate consumption. Concentrations of **(A)** OD_600_, **(B)** ethylene glycol, **(C)** glucose, **(D)** oxalate, **(E)** mevalonate. **(F)** ^13^C enrichment in mevalonate after 6 h of cultivation. **(G)** Relative isotopologue distribution in acetyl-CoA after 6 h inferred from the corresponding distribution in mevalonate. Absence of measurable ^13^C-labeling is indicated by an asterisk (*). Error bars indicate the standard error of the mean (n = 3).

Without glucose supplementation, strains inoculated at LCD could not grow and substrate consumption remained negligible, confirming the results of Mückschel and colleagues (Mückschel et al., 2012, not shown). For LCD conditions cultivated in the presence of glucose beads or 111 mM glucose, as well as HCD cultivations without glucose supply, with glucose beads or 111 mM glucose, optical density, substrate conversion and product concentrations are shown in Figure 2. In HCD cultures, complete consumption of 50 mM EG was observed after 48 h (Figure 2B). This demonstrates the advantage of *P. putida* KT2440 over *E. coli*, as in the latter strain even a ten-fold higher supply of EG (500 mM) only gave rise to a very residual substrate consumption, which was too low to be quantified (Wagner et al., 2023a). However, in the conditions where glucose was supplemented at 111 mM, EG consumption was virtually undetectable during the first 6 hours. EG uptake was initiated after glucose had been depleted from the medium indicating that the sugar inhibits EG assimilation (Figures 2B,C).

The intermediates of natural EG assimilation in *P. putida* KT2440 are GA, glycolate, glyoxylate and oxalate (Mückschel et al., 2012; Franden et al., 2018). GA and glyoxylate did not accumulate in the medium at significant concentrations (data not shown). Glycolate was only detectable at low levels except in the LCD condition containing glucose beads, where 57%–59% of the consumed EG were converted to glycolate (Supplementary Figure S2A). However, between 2.4 mM and 18.9 mM of oxalate - the product of glyoxylate oxidation, were produced in all conditions after 48 h, which represented 10%–39% of the consumed EG. The highest oxalate concentrations were observable in conditions where glucose was absent or slowly released from the feed beads (Figure 2D). A possible explanation for this observation is a limited availability of the glyoxylate-acceptor acetyl-CoA in these conditions, preventing glyoxylate assimilation through the glyoxylate shunt and causing its oxidation to oxalate.

The highest MVA concentration (0.1 mM) was observed in HCD cultures, when glucose was available at high concentrations (Figure 2E). While this represents an extremely low product yield compared to previously reported MVA production in *P*. *putida* (Yang et al., 2020; Zhang et al., 2024), the model product was mainly intended to serve as a tool to analyze the contribution of different pathways in regards to acetyl-CoA production. Hence, optimizing conversion of acetyl-CoA to MVA was initially not prioritized.

To determine how much of the produced MVA (and thus acetyl-CoA) originated from EG-dependent pathways, we supplied uniformly ^13^C-labeled EG as the carbon source and measured the ^13^C isotopologue distribution of MVA after 6 h. At this timepoint, significant EG consumption had been observed in all conditions except those where glucose was present in high concentrations. In the latter conditions, labeling data only changed marginally after 24 h, when EG was partly consumed (Supplementary Figure S2). No EG-derived MVA was observable after 6 h in LCD cultivations (Figure 2F). In the high glucose LCD condition this was caused by delayed consumption of EG. When glucose beads were used in LCD, EG was mostly converted to glycolate (57%–59%) and less than 1 µM of MVA was produced in total, leading to no detectable ^13^C incorporation.

In the HCD cultures, a maximum of 5.5% of MVA consisted of ^13^C when glucose was provided by feed beads, whereas only very small amounts of ^13^C carbon (0.1%) were detectable when glucose was supplemented at 111 mM. Thus, increased glucose availability facilitated increased acetyl-CoA and MVA production from the unlabeled carbon source (Figures 2E,F) and reduced EG consumption due to the sequential assimilation of the substrates.

When no glucose was supplemented, 3.3% of the carbon in MVA was labeled, which was surprising considering that no unlabeled substrate was added to the cultures. In this condition, the catabolic mobilization of cellular components or storage carbohydrates such as PHAs may account for sources of the unlabeled carbon in acetyl-CoA (Prieto et al., 2016; Mezzina et al., 2021). As the large fraction of unlabeled MVA in the absence of glucose could indicate potential isotopic dilution from unlabeled (storage) metabolite pools, the presented contribution of ethylene glycol assimilation pathways to acetyl-CoA formation should be viewed as a lower limit.

To characterize the ^13^C-labeling pattern of acetyl-CoA, the mass isotopologue distribution of the accumulated MVA was fit to a trinomial distribution model. This combinatorial approach assumes a spatially homogeneous cytosolic pool of acetyl-CoA, from which three acetyl-CoA molecules are randomly combined to form MVA. The contribution of background fluxes yielding MVA pathway intermediates, e.g., *via* degradation of amino acids, was assumed to be negligible during cultivation on mineral medium.

The dominant isotopologue fraction of acetyl-CoA was M+1 in all conditions (Figure 2G). When acetyl-CoA is generated *via* the engineered SAGA pathway, the only possible labeling state of acetyl-CoA is M+2, because both its carbon atoms originate from the uniformly labeled substrate EG. Therefore, the presence of M+1 acetyl-CoA arose from competing, native, EG-consuming pathways. In wild-type *P. putida* KT2440 cells, the glycerate pathway, which converts EG *via* glyoxylate, tartronate semialdehyde and glycerate to acetyl-CoA is not active (Mückschel et al., 2012; Franden et al., 2018). Alternatively, EG assimilation can produce acetyl-CoA through oxidation to GA, glycolate and glyoxylate which is taken up *via* the glyoxylate shunt (Mückschel et al., 2012; Franden et al., 2018) (Figure 1). The TCA cycle intermediates malate and oxaloacetate can then be converted to acetyl-CoA, undergoing two decarboxylation reactions (Sudarsan et al., 2014). The decarboxylation of pyruvate cleaves an EG-derived carbon atom off the carbon skeleton, yielding M+1 acetyl-CoA. Because the M+1 fraction was the most abundant isotopologue in MVA, we conclude that the natural EG assimilating pathways were far more active than the engineered SAGA pathway in strain SMP1.

### 3.2 Mutational studies identify all GA reductases in *Pseudomonas putida* KT2440 and indicate presence of unknown GA dehydrogenases

**FIGURE 3:**
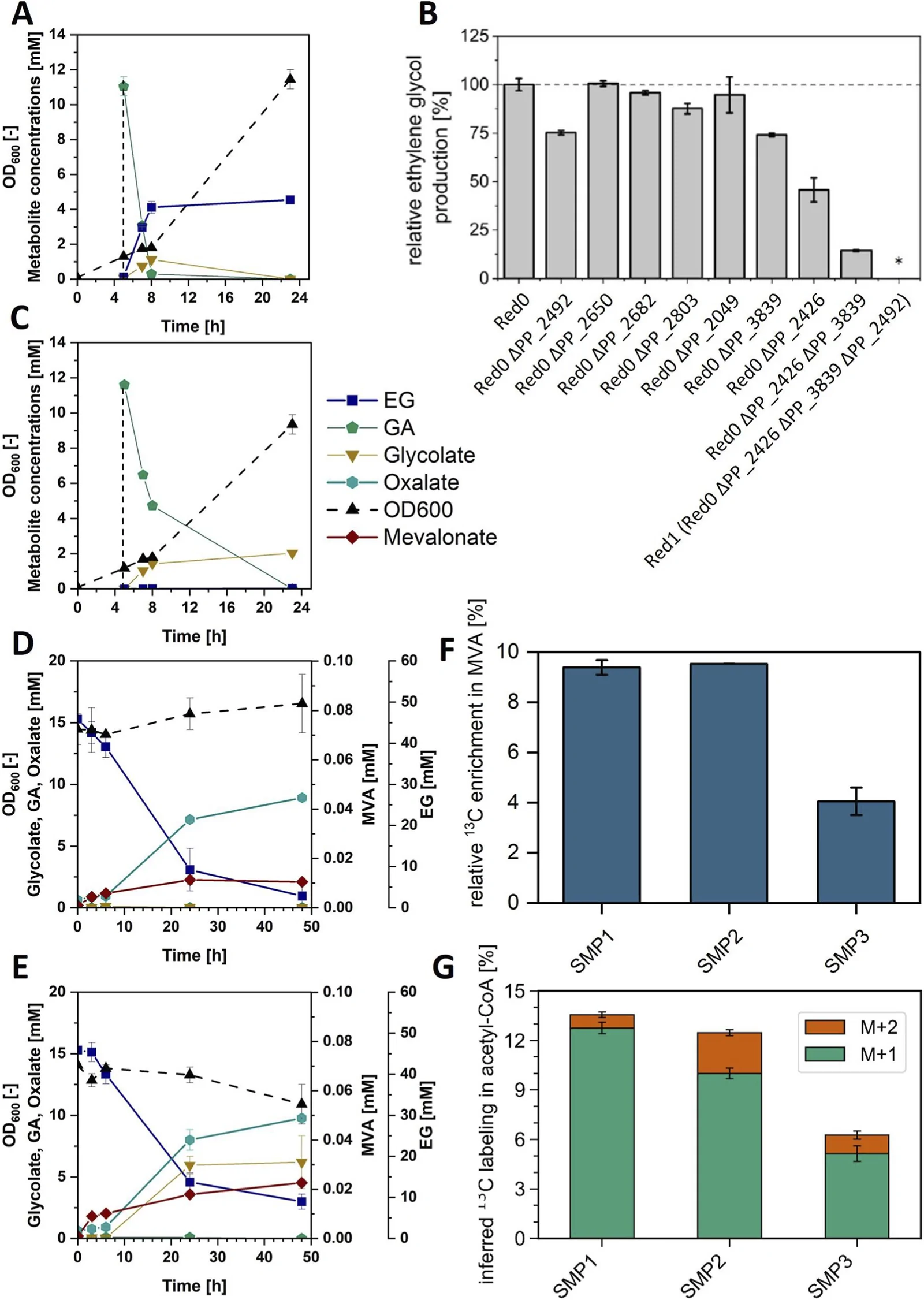
Identification of glycolaldehyde (GA) reductases in *Pseudomonas putida* KT2440 and assessment of the impact on SAGA pathway flux. Red0 (*Pseudomonas putida* KT2440 *ΔaldB-I ΔaldB-II*) and derived strains were cultivated in M9 mineral medium containing 111 mM glucose at 30 °C. Cells were inoculated with an OD_600_ of 0.2 and 10 mM GA was added in the early exponential growth phase (OD_600_ ∼ 1.2). **(A)** Growth and GA conversion of strain Red0. **(B)** Relative ethylene glycol (EG) formation of Red0-derived strains 3 h after GA addition. Values are normalized to EG formation detected for Red0, 3 h after GA addition (2.96 mM). Absence of measurable concentrations is indicated by an asterisk (*). **(C)** Growth and GA conversion of strain Red1 (Red0 ΔPP_2426 ΔPP_3839 ΔPP_2492). Impact on SAGA pathway performance was assayed in strains SMP1 (*Pseudomonas putida* KT2440–Ec.atoB-Lc.mvaS-Lc.mvaE-Ec.KdsD(c-opt)-Ec.Rpe-Ec.Pta + pBTL2-Ec.FsaA(c-opt)-Ca.Pkt(c-opt)), SMP2 (SMP1 *ΔaldB-I ΔaldB-II*) and SMP3 (SMP2 ΔPP_2426 ΔPP_3839 ΔPP_2492) cultivated in M9 medium supplemented with glucose releasing beads and 50 mM ^13^C_2_-EG. Cells were inoculated in high-cell-density (OD_600_ ∼ 14). Growth, substrate conversion and product formation of **(D)** SMP2 and **(E)** SMP3. **(F)** Enrichment of mevalonate (MVA) in ^13^C after 24 h of cultivation. **(G)** Relative isotopologue distribution in acetyl-CoA after 24 h inferred from the corresponding distribution in MVA. Error bars indicate standard error of the mean (n ≥ 2)

The Ara5P aldolase enzyme Ec. FsaA has a high *K*_m_ for GA [62.8 mM for GA as donor substrate (Garrabou et al., 2009)] compared to annotated GA dehydrogenases [e.g., Ec. AldA, *K*_m_ = 0.14–0.21 mM (Rodríguez-Zavala et al., 2006; Lachaux et al., 2019)]. This may explain why EG was preferentially assimilated *via* the natural pathway in strain SMP1. Therefore, we tried to increase carbon flux through the SAGA pathway by increasing the intracellular availability of GA. To this end, the two known GA dehydrogenases AldB-I (PP_0545) and AldB-II (PP_2680) (Mückschel et al., 2012; Franden et al., 2018) were deleted in *P. putida* KT2440 yielding strain Red0. GA metabolism in this strain was characterized by adding 10 mM GA to a culture which grew exponentially on glucose. As shown in Figure 3A, strain Red0 rapidly consumed the aldehyde within 3 h after addition. Approximately 40% of GA were recovered in the form of EG, while 60% were assimilated *via* the natural pathway as witnessed by the transient accumulation of glycolate. From these observations we concluded that *P. putida* KT2440 possesses additional GA dehydrogenases. Additionally, the formation of EG shows that the strain also operates efficient GA reductases.

To identify enzyme candidates catalyzing the reduction of GA, amino acid sequences of known GA reductases [Ec.YqhD, Ec. FucO and Go. Gox0313 (Alkim et al., 2015; Zhang et al., 2015; Jo et al., 2022)] were aligned against the proteome of *P. putida* KT2440 (https://blast.ncbi.nlm.nih.gov). For each of the three reference GA reductases, two to three candidate enzymes were selected based on high sequence similarity (see Supplementary Table S3).

Genes encoding potential GA reductases were individually deleted in the parent strain Red0. Reduction of GA to EG by these strains was quantified under the same conditions as applied for Red0. While the parent strain Red0 produced 2.96 mM EG, individual deletion of PP_2492 (*yqhD*, Ec-YqhD ortholog), PP_3839 (*adhP*) or PP_2426 (*calA*) (Go-Gox0313 orthologs) significantly reduced the conversion of GA to EG, with the PP_2426 deletion having the largest effect (55% reduced EG production, Figure 3B). In successive steps, the PP_3839 and PP_2492 genes were deleted in addition to PP_2426, which completely prevented the reduction of GA to EG by the resulting strain Red1 (Figure 3C). While the GA uptake rate of the GA reductase-deficient strain Red1 was found to be reduced compared to the parental strain Red0, the aldehyde was still completely consumed through the oxidative pathway (see Figures 3A,C).

### 3.3 Impact of altered GA metabolism on SAGA pathway function

To test whether eliminating the two known GA dehydrogenases (AldB-I and AldB-II) reduces the flux through the natural pathways, both corresponding genes were deleted in the production strain SMP1 resulting in strain SMP2. Furthermore, it was analyzed whether the additional deletion of all newly identified GA reductases in strain SMP3 had an impact on conversion of EG to acetyl-CoA.

As shown above (Figure 2G), HCD cultivation in combination with supplementation of glucose-releasing beads and 50 mM ^13^C_2_-EG supported the highest incorporation of labeled carbon into MVA and provided the largest fraction of acetyl-CoA produced through the SAGA pathway (1.3%). Consequently, the same cultivation strategy was retained for the comparison of strains SMP2 and SMP3. Both strains consumed EG at similar rates during the 48 h experiment (Figures 3D,E). Although SMP2 and SMP3 both lacked the two known GA dehydrogenases AldB-I and AldB-II, the strains still produced approximately 9 mM oxalate. Because oxalate is an end-product of natural EG assimilation (Figure 1; Mückschel et al., 2012), this accumulation is another indicator that additional enzymes with GA dehydrogenase activity exist in *P. putida* KT2440, an organism which is known to contain a vast repertoire of aldehyde dehydrogenases with broad substrate spectrum (Coitinho et al., 2016; Shi et al., 2022). Glycolate accumulation differed markedly between the two strains: For SMP2 only trace amounts (<0.1 mM) were detectable (Figure 3D), whereas 6.19 mM were present in the medium of SMP3 after 48 h (Figure 3E). This discrepancy is surprising, considering the downstream oxidation product oxalate was formed at comparable levels in both strains. Moreover, glycolate also accumulated in strain Red1 but not in strain Red0 during the reductase screening (Figures 3A,C), indicating that the deletion of GA reductases substantially alters glycolate metabolism.

Strain SMP3 produced roughly twice as much MVA as strain SMP2 after 48 h (0.023 mM vs. 0.010 mM; Figures 3D,E). As a significant amount of EG was consumed in both SMP2 and SMP3 after 24 h, we analyzed ^13^C labeling in MVA at that timepoint and compared it to SMP1. Only 4.0% of MVA produced by SMP3 contained EG-derived carbon, compared with 9.5% for SMP2 (Figure 3F). This relative drop in label incorporation indicates that the higher MVA yield of SMP3 was not due to an improved conversion of EG to acetyl-CoA.

Comparing strain SMP2 to the base strain SMP1, the overall ^13^C enrichment in MVA was essentially unchanged (9.4% in SMP1 vs. 9.5% in SMP2). However, the M+1 isotopologue fraction of acetyl-CoA decreased by 22% (12.7% to 10.0%, Figure 3G) following the deletion of *aldB-I* and *aldB-II* (SMP2). In contrast, the M+2 fraction increased by a factor of three (0.8% to 2.5%), indicating that both knockouts positively affected flux through the engineered SAGA pathway. In contrast, deletion of the 3 GA reductases did not further enhance SAGA pathway flux in strain SMP3. Since GA reduction yields EG, this reaction does not divert carbon away from the SAGA pathway. The reduced competition for GA caused by the triple deletion evidently did not increase flux across the reaction catalyzed by FsaA. Therefore, strain SMP2 was retained as the platform for further pathway optimization.

### 3.4 Abolishing PQQ-dependent glucose assimilation enables simultaneous uptake of glucose and ethylene glycol

During the cultivation of SMP1, EG consumption was strongly inhibited when the cultivation medium contained a significant concentration of glucose. In *E. coli*, the presence of a co-substrate that can supply the GA acceptor GA3P is essential for SAGA pathway function, presumably because regeneration of GA3P *via* the SAGA pathway is insufficient (Wagner et al., 2023a). To circumvent a potential limitation of GA3P, we aimed at enabling simultaneous assimilation of EG and the co-substrate glucose.

The simultaneous presence of multiple carbon sources can trigger carbon catabolite repression (CCR) (Moreno and Rojo, 2024), a phenomenon enabling bacteria to prioritize energetically favorable substrates. Canonical CCR mechanisms can involve the downregulation of catabolic genes required for the metabolism of less preferred carbon sources by transcriptional repression (Rojo, 2010). To determine whether transcriptional repression mediates the sequential consumption of glucose and EG, we constructed a *P. putida* KT2440 reporter strain, where the gene encoding the primary EG oxidizing enzyme, that is, the PQQ-dependent alcohol dehydrogenase PedE, was replaced with a sequence encoding the fluorescent protein SYFP2 (Frazão et al., 2018).

**FIGURE 4:**
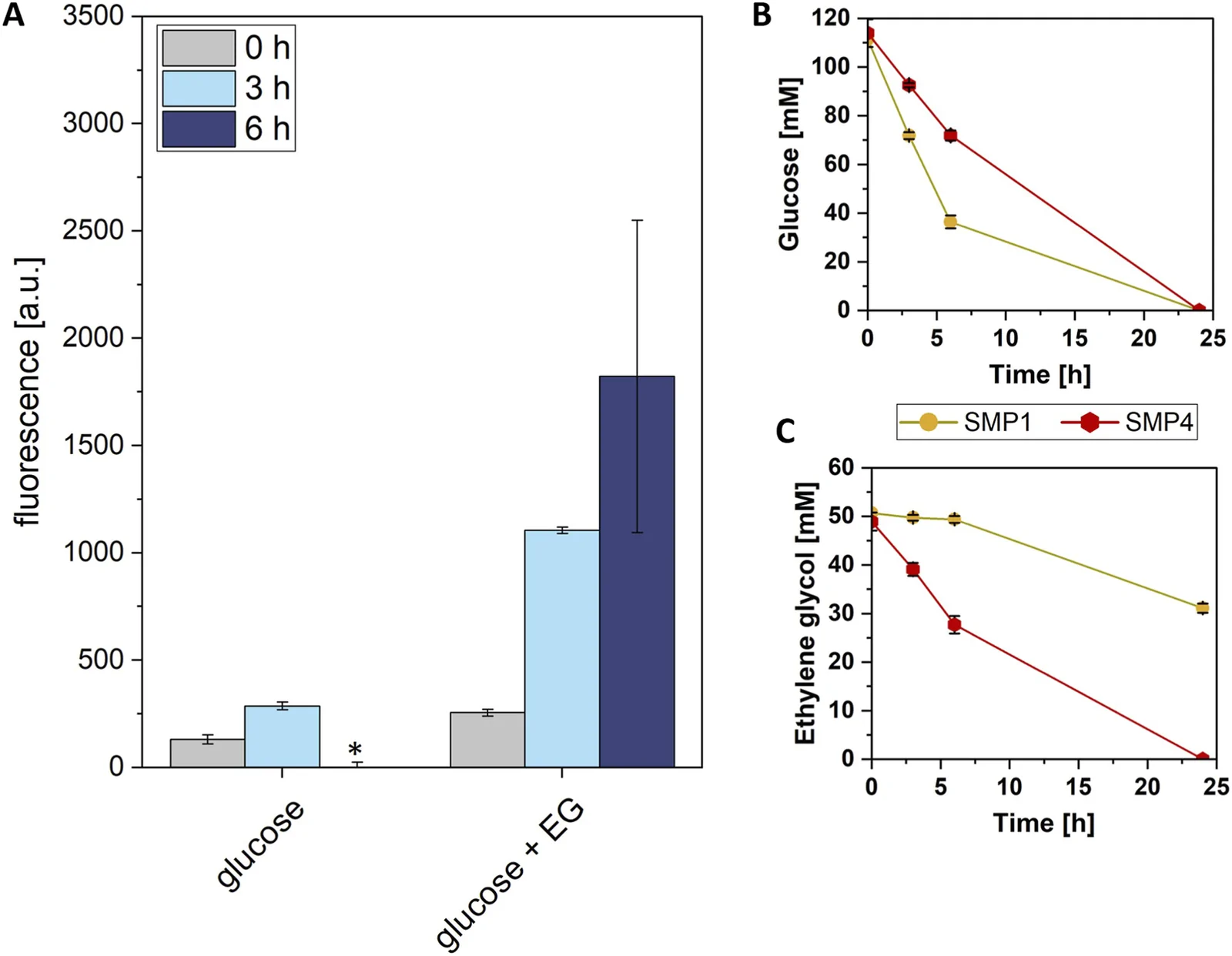
Uptake of glucose and EG in wildtype and mutant cells. **(A)** Fluorescence intensity of *Pseudomonas putida ΔpedE*::syfp2 cells growing on pure glucose or a mixture of glucose and EG. Glucose and EG concentrations were 33 mM and 320 mM, respectively. Fluorescence values represent the difference between fluorescence intensity of *Pseudomonas putida* KT2440 *ΔpedE*::syfp2 and the background determined using *Pseudomonas putida* KT2440 wildtype. Absence of fluorescence is indicated by an asterisk (*). **(B)** Glucose and **(C)** EG consumption by strains SMP1 (*Pseudomonas putida* KT2440–Ec.atoB-Lc.mvaS-Lc.mvaE-Ec.KdsD(c-opt)-Ec.Rpe-Ec.Pta + pBTL2-Ec.FsaA(c-opt)-Ca.Pkt(c-opt)) and SMP4 (SMP1 *Δgcd*). Strains were inoculated at OD_600_ ∼ 12 and cultivated in M9 medium containing 111 mM glucose and 50 mM EG. Error bars indicate standard error of the mean (n ≥ 2).

The reporter strain (Δ*pedE*::SYFP2) and a wildtype control were cultivated in M9 medium containing 33 mM glucose with or without the additional supplementation of 320 mM EG. The differences in fluorescence intensities between the reporter strain and the wildtype strain are shown in Figure 4A. Only when EG was present, the reporter strain exhibited a significant difference in fluorescence compared to the wildtype. As this difference was already observable after 3 h, we conclude that glucose does not prevent EG-mediated induction of *pedE*. Thus, delayed EG assimilation in the presence of glucose must be caused by a different mechanism.

Since glucose is preferentially assimilated *via* the PQQ-dependent glucose dehydrogenase [encoded by *gcd* (PP_1444) (Sudarsan et al., 2014; Nikel et al., 2015)], and EG assimilation proceeds *via* PQQ-dependent PedE, we speculated that the sequential uptake of both substrates might be caused by limitation of the common cofactor PQQ. To test whether co-metabolization of EG and glucose could be achieved by increasing the availability of PQQ for PedE, *gcd* was deleted in strain SMP2. The resulting strain SMP4 was still able to grow on glucose, due to the presence of an alternative glucose-assimilating pathway which proceeds *via* phosphorylation to glucose 6-phosphate [catalyzed by glucokinase Glk (Nikel et al., 2015)]. The strains SMP1 and SMP4 were inoculated at OD ≈ 12 and their substrate consumption was analyzed in M9 medium containing 111 mM glucose and 50 mM EG (Figures 4B,C). SMP4 consumed EG rapidly (21.22 mM within 6 h) in the presence of high glucose concentrations, whereas SMP1 showed only minimal EG uptake (1.31 mM) in the same period. These results suggest that preventing PQQ consumption in the Gcd-dependent glucose oxidation alleviates PQQ-limitation of the PedE reaction, thereby enabling simultaneous uptake of both substrates.

### 3.5 Heterologous expression of *rpe* and *kdsD* genes from *Escherichia coli* improves SAGA pathway function

*In-vivo* studies of the SAGA pathway in *E. coli* demonstrated that overexpression of isomerases converting Ara5P into Xylu5P is essential for the improvement of SAGA pathway performance (Wagner et al., 2023a). Based on these observations, we investigated the effect of overexpressing all SAGA pathway enzymes by chromosomally integrating the *E. coli* Ara5P isomerase *kdsD* and ribulose-phosphate 3-epimerase *rpe* into strain SMP4, generating strain SMP7. To assess the requirement to express phosphoketolase and arabinose-5P aldolase activities in *P. putida* KT2440, strains were constructed which carried the same genetic modifications as SMP4 but did not express either *Ca. pkt* (SMP5) or *Ec. fsaA* (SMP6). All strains were inoculated at HCD (OD_600_ ≈ 12) and cultivated in M9 medium containing 111 mM glucose and 50 mM ^13^C_2_-EG.

**FIGURE 5:**
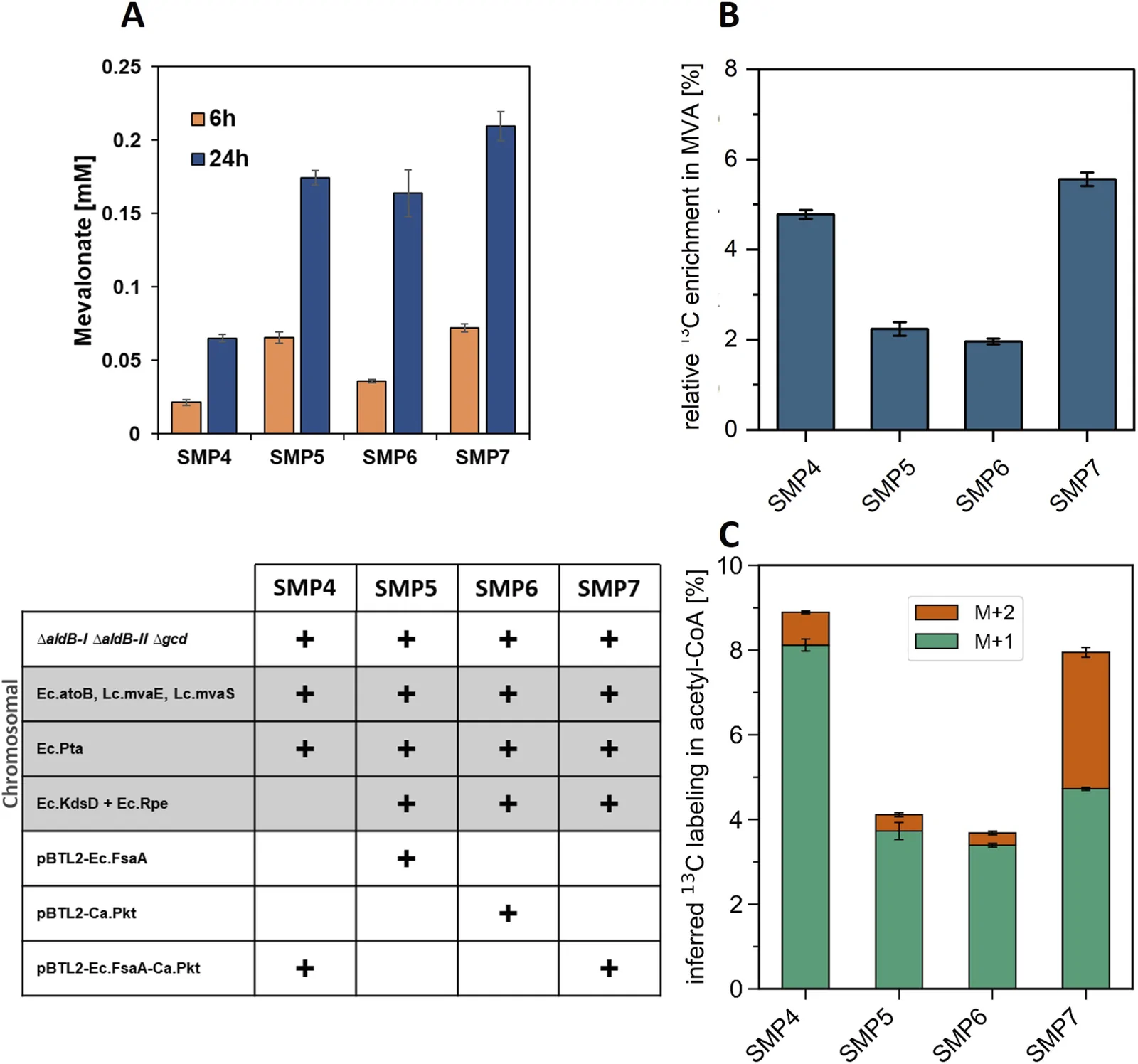
Investigating the effect of expressing different combinations of SAGA genes on mevalonate (MVA) formation from EG. SMP4-7 were inoculated to OD ∼12 and cultivated in M9 medium supplemented with 50 mM ^13^C_2_-EG and 111 mM glucose at 30 °C. **(A)** Extracellular MVA accumulation after 6 h and 24 h. **(B)** Relative ^13^C enrichment in MVA after 6 h. **(C)** Relative isotopologue distribution in acetyl-CoA after 6 h inferred from the corresponding distribution in MVA. Error bars indicate standard error of the mean (n = 2).

Strains harboring the heterologous *Ec. kdsD* and *Ec. rpe* genes (SMP5, SMP6 and SMP7) displayed a more than two-fold increase in MVA production relative to SMP4 after 24 h of cultivation (Figure 5A). The most pronounced effect was observed for strain SMP7 in which all SAGA pathway genes were overexpressed, which accumulated 0.21 mM MVA after 24 h.

The ^13^C-incorporation in MVA in the different pathway settings was compared after 6 h, since both glucose and EG were still present in substantial concentrations at this time during the cultivation (Supplementary Figure S3). When either *Ca. pkt* or *Ec. fsaA* expression was omitted (strains SMP5 and SMP6, respectively), the M+2 fraction in acetyl-CoA represented only 0.38% and 0.28%, showing that expression of these heterologous enzymes was crucial for efficient functioning of the SAGA pathway (Figure 5C). A similar trend was observable in the absence of heterologous *kdsD* and *rpe*. Here the M+2 fraction amounted to 0.78%. The highest relative ^13^C enrichment in MVA was observed in the strain overexpressing all SAGA pathway enzymes (SMP7, 5.6%, Figure 5B). Compared to strain SMP4, strain SMP7 exhibited a 4.2-fold higher contribution of the SAGA pathway to the total acetyl-CoA production (3.2%). Collectively these results show that the simultaneous overexpression of all SAGA pathway genes maximizes both MVA yield and SAGA pathway flux. Hence, SMP7 was selected as the platform for subsequent strain optimization work.

### 3.6 Native assimilation *via* glycolate is the major pathway diverting GA away from the SAGA pathway

**FIGURE 6:**
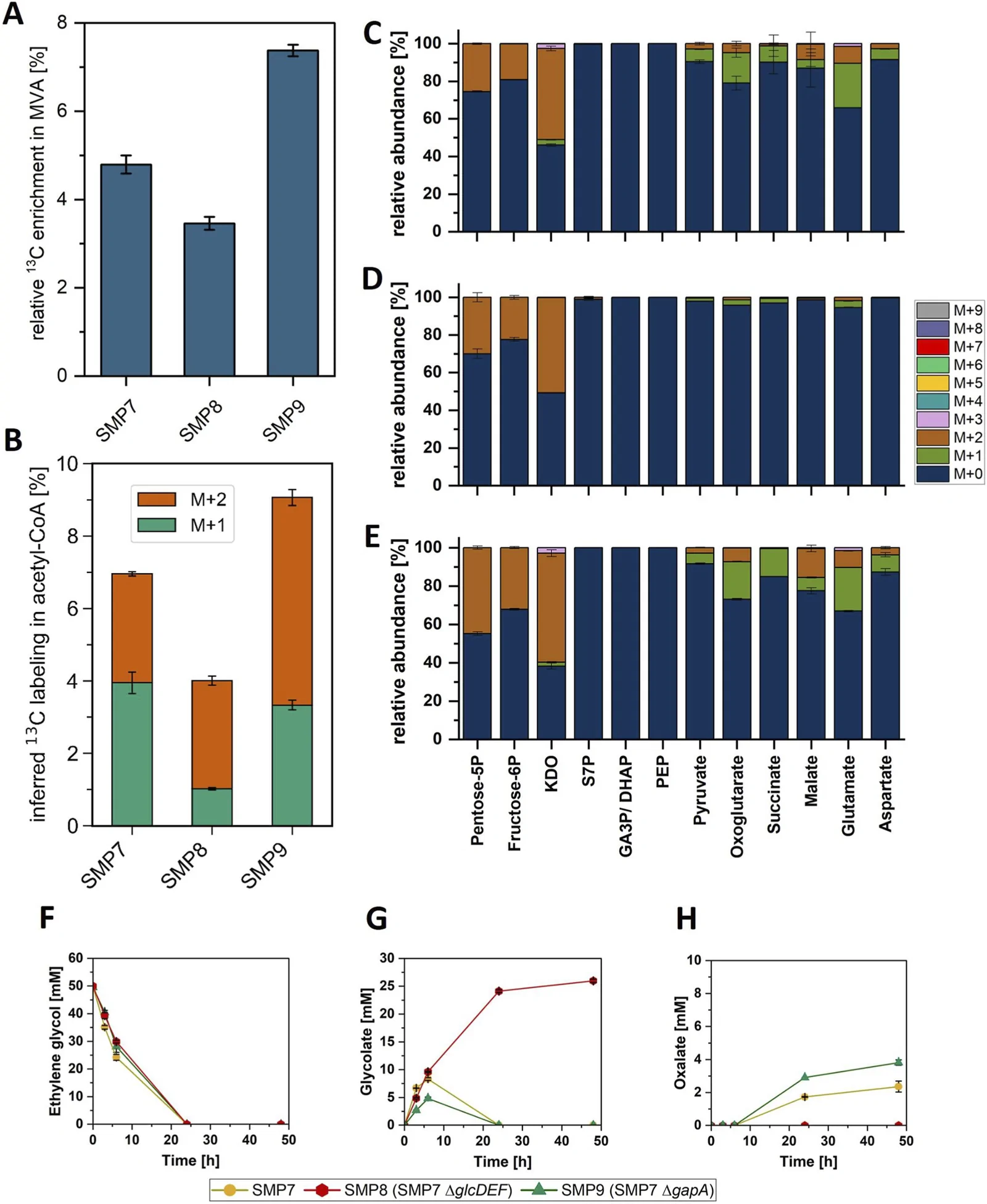
Formation of mevalonate (MVA) and intracellular metabolites from ethylene glycol (EG) in *Pseudomonas putida* strains engineered for increased SAGA pathway activity or decreased flux through competing pathways. Cells were inoculated at OD_600_ ∼ 9 and cultivated in M9 medium supplemented with 111 mM glucose and 50 mM ^13^C_2_-EG at 30 °C. Strains are derived from strain SMP7 (*Pseudomonas putida* KT2440 *ΔaldB-I ΔaldB-II Δgcd*- Ec. atoB-Lc.mvaS-Lc.mvaE-Ec.KdsD(c-opt)-Ec.Rpe-Ec.Pta + pBTL2-Ca.Pkt(c-opt)-Ec.FsaA(c-opt)). **(A)** ^13^C enrichment in MVA of SMP7, SMP8 (SMP7 *ΔglcDEF*) and SMP9 (SMP7 *ΔgapA*) after 6 h of cultivation. **(B)** Relative isotopologue distribution in acetyl-CoA after 6 h inferred from the corresponding distribution in MVA. Relative abundance of ^13^C isotopologues in extracts of intracellular metabolites after 6 h of cultivation of strain **(C)** SMP7, **(D)** SMP8 and **(E)** SMP9. Pentose 5-phosphates (which are D-Arabinose 5-phosphate, D-Ribulose 5-phosphate, D-Xylulose 5-phosphate and D-ribose 5-phosphate) and GA3P/DHAP are shown as pools since they could not be separated by LC/MS. Concentrations of **(F)** EG, **(G)** Glycolate and **(H)** Oxalate measured in the supernatant (extracellular) of strains SMP7, SMP8 and SMP9. Error bars indicate standard of the mean (n = 2). Abbreviations: DHAP, dihydroxyacetone phosphate; GA3P, glyceraldehyde 3-phosphate; KDO, 2-Keto-3-Deoxy-D-Mannooctanoic acid; PEP, phosphoenolpyruvate; S7P, sedoheptulose 7-phosphate.

All strains examined so far accumulated a significant amount of M+1 labeled acetyl-CoA. As noted above, this isotopologue cannot be a product of the SAGA pathway. A plausible source of the M+1 isotopologue is the EG assimilation route native to *P. putida* KT2440, where EG is converted to glyoxylate, which can then enter the TCA cycle through the glyoxylate shunt (Figure 1). Deletion of both genes encoding known GA dehydrogenases (*aldB-I, aldB-II*) did not abolish flux through this pathway. Therefore, we investigated whether eliminating the subsequent oxidation of glycolate to glyoxylate, catalyzed by glycolate oxidase GlcDEF, would completely block this route. To this end, the parent strain SMP7, and its *ΔglcDEF* derivative (SMP8), were inoculated to OD_600_ ≈ 9 and cultivated in M9 medium supplemented with 111 mM glucose and 50 mM ^13^C_2_-EG. The resulting ^13^C-labeling patterns in metabolites of the central metabolism were compared after 6 h of cultivation when the cells were neither limited for glucose nor for EG (Figure 6F; Supplementary Figure S4).

The deletion of *glcDEF* (strain SMP8) significantly diminished the M+1 isotopologue in acetyl-CoA, resulting in a 74% reduction relative to the *glcDEF*-competent strain SMP7 (Figure 6B). The observable 28% reduction in ^13^C enrichment in MVA of SMP8 compared to SMP7 (Figure 6A) can be predominantly attributed to this reduction in M+1 labeling, as the M+2 fraction was identical in both strains, indicating that SAGA pathway activity was not affected. To assess how this deletion reshaped carbon flow, we measured isotopologue distributions in central, intracellular metabolites (Figures 6C,D). In both SMP7 and SMP8, 25% and 30% of the pentose 5-phosphate pool (Ara5P, Ribu5P, Xylu5P and D-ribose 5P) were labeled M+2 while the remaining 75% and 80% were unlabeled. Approximately 50% of the cell wall precursor 3-deoxy-α-D-manno-2-octulosonate (KDO) (Smyth and Marchant, 2013) contained two labeled carbon atoms in both strains. Presumably its precursor Ara5P contained the same M+2 fraction. Combined with the fact that the M+2 fractions contributed significantly to ^13^C enrichment of acetyl-CoA (3.0% in both strains, Figure 6B), these findings confirm that the SAGA reaction sequence was active *in-vivo* and played a significant role in pentose 5-phosphate biosynthesis. A pronounced difference between the strains emerged in the isotopologue distribution of TCA cycle intermediates. In SMP7 the intermediates malate, oxoglutarate and succinate as well as pyruvate contained significant amounts of labeled carbon which was likely produced by the addition of fully labeled glyoxylate to unlabeled acetyl-CoA *via* the glyoxylate shunt. Since M+1 was the major isotopologue in all these intermediates except malate, the latter was likely decarboxylated twice yielding acetyl-CoA. During this conversion, one of the cleaved carboxyl groups contains a ^13^C isotope, resulting in the formation of M+1 labeled acetyl-CoA.

By contrast, the *glcDEF* deficient strain SMP8 showed virtually no labeling in these central metabolites, indicating that almost no EG-derived carbon was channeled through the glyoxylate shunt. Instead SMP8 accumulated mainly glycolate (24.1 mM at 24 h, Figure 6G) which amounts to 48% of the consumed EG (Figure 6F). In contrast, glycolate production peaked at 6 h in strain SMP7 (4.8 mM) but was fully re-consumed by 24 h. Furthermore, SMP7 accumulated 2.9 mM oxalate after 24 h (Figure 6H), whereas oxalate was absent in SMP8, which is another indicator for successful interruption of the native EG assimilation sequence. Collectively these results demonstrate that disabling the native EG assimilation pathway nearly abolishes the incorporation of EG derived carbon into TCA metabolites, greatly reducing the formation of M+1 MVA and thus confirming that this pathway diverts a major carbon fraction away from the SAGA pathway.

### 3.7 Deletion of a major glyceraldehyde-3P dehydrogenase increases SAGA pathway flux

Simultaneous assimilation of EG and glucose markedly improved flux through the engineered SAGA pathway. A possible explanation for this finding is that presence of the co-substrate glucose increased the availability of the GA-acceptor GA3P, thereby facilitating the formation of Ara5P at the entry of the SAGA pathway (Figure 1). In an attempt to raise intracellular GA3P levels in the producer strain, we deleted *gapA* (PP_1009) in strain SMP7, which is one of the two major GA3P dehydrogenases oxidizing GA3P to glycerate-1,3-biphosphate (Nikel et al., 2014). The resulting strain SMP9 showed a 48% reduction in growth rate compared to the *gapA* competent strain SMP7 (Supplementary Table S4), indicating that the remaining GA3P dehydrogenases could not fully compensate for the loss of *gapA*. The EG conversion of the resulting strain SMP9 was characterized under the same conditions as the SMP7 reference strain. Analysis of the labeling pattern of the MVA pool revealed that deletion of *gapA* increased the M+2 fraction of acetyl-CoA by 91% (Figure 6E). Furthermore, the intracellular pentose 5-phosphate pool in SMP9 exhibited a 76% larger M+2 isotopologue fraction than in SMP7 and the M+2 labeling of Ara5P derived cell wall precursor KDO increased by 17% (Figure 6F). These results suggest that enhancement of SAGA pathway can be attributed to the higher intracellular availability of GA3P.

### 3.8 Plasmid-borne overexpression of the mevalonate pathway strongly increases product yield

All strains examined so far accumulated less than 0.3 mM MVA when cultivated in M9 medium containing 111 mM glucose and 50 mM EG. In these strains, the MVA pathway capacity was provided by three chromosomally integrated genes: *Ec. atoB*, *Lc. mvaE*, *Lc. mvaS*. To assess whether increasing gene copy number could enhance MVA production, all three genes were additionally cloned into the medium copy pBTL2 plasmid, which also carried the SAGA pathway genes *Ec. fsaA* and *Ca. pkt*. The original plasmid construct pBTL2-Ec.FsaA-Ca.Pkt, present in strain SMP9, was replaced by pBTL2-Ec.FsaA-Ca.Pkt-Ec.atoB-Lc.mvaS-Lc.mvaE, generating strain SMP10. SMP10 was inoculated at OD_600_ ≈ 9 and cultivated in M9-medium supplemented with 50 mM uniformly labeled ^13^C_2_-EG and either 111 mM glucose or glucose-releasing beads.

**FIGURE 7:**
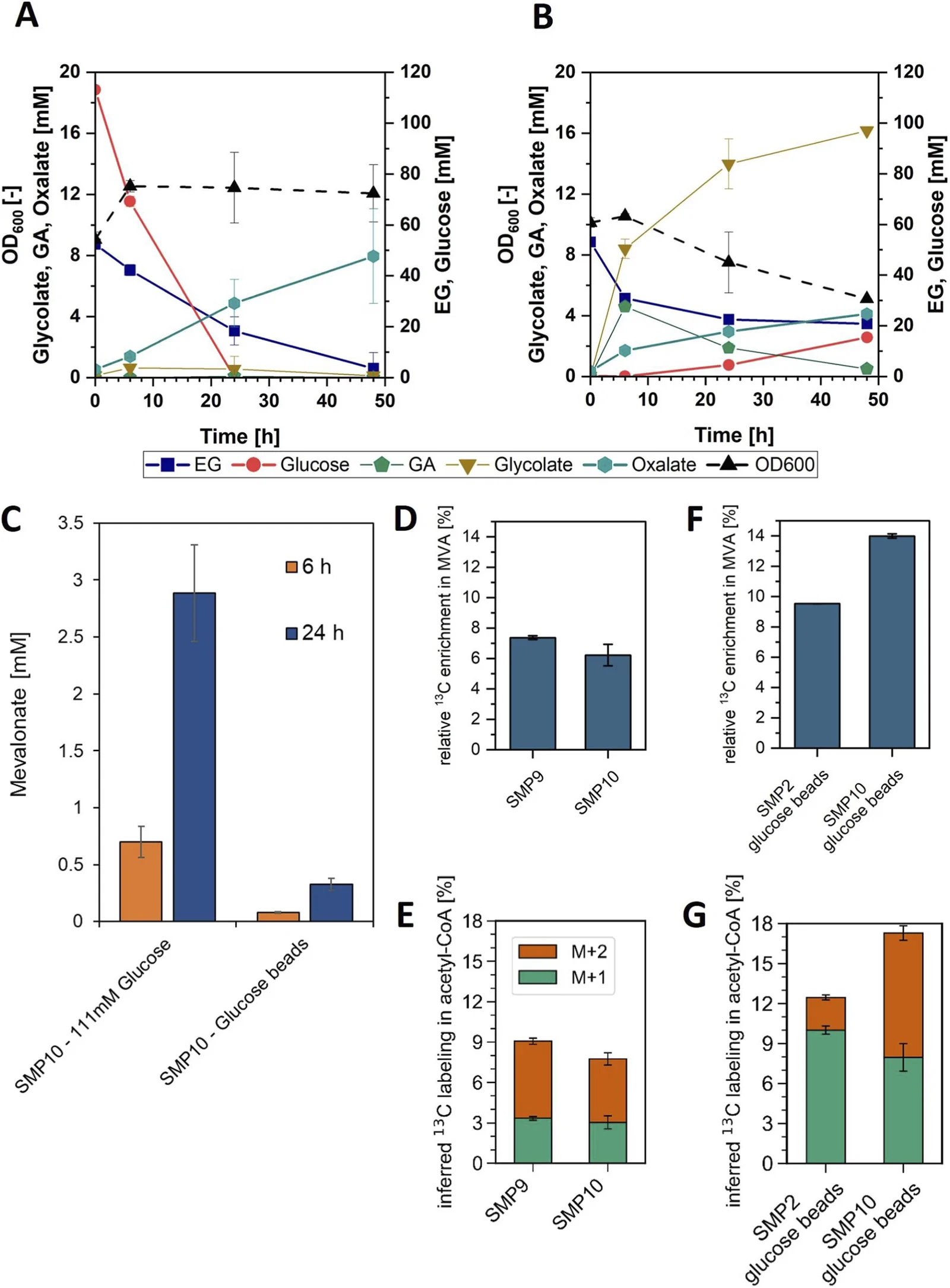
Conversion of EG into mevalonate (MVA) by strain SMP10. (*Pseudomonas putida* KT2440 *ΔaldB-I ΔaldB-II Δgcd ΔgapA*–Ec.atoB.Lc.mvaS-Lc.mvaE-Ec.KdsD(c-opt)-Ec.Rpe-Ec.Pta + pBTL2- Ec. FsaA(c-opt)-Ca.Pkt(c-opt)-Ec.atoB-Lc.mvaS-Lc.mvaE) engineered for increased MVA production. Cells were inoculated at OD_600_ ∼ 9 and cultivated in M9 medium supplemented with 50 mM ^13^C_2_-EG and either 111 mM glucose or glucose releasing beads at 30 °C. Substrate consumption, biomass and metabolite production of SMP10 cultivated with **(A)** 111 mM glucose or **(B)** glucose releasing beads. **(C)** Production of MVA after 6 h and 24 h **(D)** ^13^C-enrichment in MVA of SMP9 (*Pseudomonas putida* KT2440 *ΔaldB-I ΔaldB-II Δgcd ΔgapA*–Ec.atoB.Lc.mvaS-Lc.mvaE-Ec.KdsD-Ec.Rpe-Ec.Pta + pBTL2- Ec. FsaA-Ca.Pkt) and SMP10 after 6 h. **(E)** Relative isotopologue distribution in acetyl-CoA of SMP9 and SMP10 after 6 h, inferred from the corresponding distribution in MVA. **(F)** ^13^C-enrichment in MVA after 24 h of SMP2 and SMP10 cultivated with glucose releasing beads. **(G)** Relative isotopologue distribution in acetyl-CoA of SMP2 and SMP10 cultivated with glucose releasing beads after 24 h, inferred from the corresponding distribution in MVA. Error bars indicate standard error of the mean (n ≥ 2).

When cultivated with 111 mM glucose, SMP10 fully consumed the co-substrate within 24 h and nearly depleted the supplied EG after 48 h (Figure 7A). Oxalate was the major by-product, accumulating to approximately 8 mM after 48 h. The expression of the MVA cassette from plasmid pBTL2 in strain SMP10 resulted in a 44-fold increase in MVA production after 24 h (2.88 mM, Figure 7C), compared to chromosomal expression alone in strain SMP9. This enhancement is consistent with the elevated plasmid copy number [reported at 30 ± 7 copies in *P. putida* KT2440 (Cook et al., 2018)]. Despite the higher absolute product concentration, the relative ^13^C-enrichment of MVA, as well as the isotopologue distribution in acetyl-CoA remained largely unchanged after 6 h (Figures 7D,E), indicating that the contribution of acetyl-CoA-generating pathways was not affected by increased downstream conversion. This supports the use of MVA as a reliable proxy for comparing acetyl-CoA production routes.

To assess whether the engineered increase in GA3P supply also has a positive effect on flux through the SAGA pathway at low glucose uptake rates, SMP10 was cultivated with 50 mM EG and glucose-releasing beads which deliver approximately 14 mM of glucose over 24 h. This represents a 75% reduction in carbon supply compared to the high-glucose condition (14 mM of C_6_ + 50 mM of C_2_ vs. 111 mM of C_6_ + 50 mM of C_2_). One notable effect of the continuously low glucose concentration was a gradual decline in cell viability throughout the cultivation, as indicated by the continuous drop in OD_600_ (Figure 7B). As a result, EG consumption stalled after 24 h, and glucose accumulated to 15.4 mM by 48 h, an outcome not previously observed in glucose-bead cultivations.

Under low-glucose conditions, MVA production dropped to 0.33 mM after 24 h, corresponding to an 89% reduction compared to the high-glucose condition (Figure 7C).

The ^13^C-labeling was compared to strain SMP2, which did not contain *gcd* or *gapA* deletions and had been cultivated under the same conditions. After 24 h, when both strains had consumed significant amounts of EG, ^13^C-enrichment in MVA revealed a 47% higher proportion of labeled carbon in MVA in SMP10 than in SMP2 (Figure 7F). This coincided with a 3.8-fold increase in the M+2 fraction of acetyl-CoA (Figure 7G), indicating that 9.3% of acetyl-CoA were formed *via* the SAGA pathway. Therefore, enabling simultaneous glucose and EG consumption, as well as increasing intracellular availability of GA3P increases flux through the SAGA pathway also when glucose is continuously supplied at low concentrations.

## 4 Conclusion

In *E. coli,* the SAGA pathway performed poorly when EG was used as the substrate (Wagner et al., 2023a). Significant pathway flux could only be established by supplying GA, the product of the initial oxidation reaction. The cause of this limitation is the thermodynamically unfavorable NAD(P)-dependent oxidation of EG [ΔrG′° = +23.7 kJ mol^−1^, pH 7 (Noor et al., 2014)] and the insufficient affinity of the downstream enzyme FsaA for GA, which could not remove GA efficiently enough to shift the reaction balance towards GA formation. In *P. putida*, the native PQQ-dependent dehydrogenases PedE and PedH (Mückschel et al., 2012; Wehrmann et al., 2020) are capable of oxidizing EG with a favorable ΔrG′° = −42.4 kJ mol^−1^. Accordingly, our results demonstrated that installing the SAGA pathway in *P. putida* KT2440 enabled efficient EG consumption.

During implementation of the SAGA pathway in *E. coli* it was shown that presence of a co-substrate increases carbon flux through the synthetic pathway, presumably by augmenting the availability of the GA acceptor GA3P (Wagner et al., 2023a). In our first attempts to implement a similar strategy for *P. putida*, we observed that EG was only assimilated after the co-substrate glucose was entirely consumed. After showing that this sequential substrate uptake was not due to a transcriptional repression of PedE, we enabled simultaneous uptake of both substrates by deleting the PQQ-dependent glucose dehydrogenase *gcd*, which alleviated the competition of PedE and Gcd for the common cofactor and enhanced carbon flux through the SAGA pathway.

*Pseudomonas putida* possesses a natural pathway which channels GA to glycolate and glyoxylate, ultimately yielding acetyl-CoA *via* the glyoxylate shunt. Although this route forms acetyl-CoA, it is carbon-inefficient, as two consecutive decarboxylation reactions are required (Franden et al., 2018; Wagner et al., 2023b). Carbon tracing experiments using uniformly ^13^C-labeled EG allowed us to quantify the relative contributions of the native EG-assimilation pathway, the engineered SAGA pathway, and unlabeled co-substrates to the formation of acetyl-CoA. Integration of all essential SAGA enzymes together with the MVA operon yielded detectable MVA production but the contribution of the SAGA pathway to acetyl-CoA formation remained negligible (0.8%). Deletion of the two known GA dehydrogenases (*aldB-I* and *aldB-II*) reduced flux through the natural pathway and increased the contribution of the synthetic pathway to acetyl-CoA formation to 2.5%. Additionally, we were able to identify all three GA-reductases in *P. putida* KT2440 (PP_2426, PP_3839, PP_2492), the deletion of which eliminated the conversion of GA to EG. However, their deletion gave no additional benefit to SAGA pathway activity.

Increasing the availability of the GA acceptor molecule GA3P by deleting GA3P dehydrogenase *gapA* had a strong positive impact on carbon flux through the SAGA pathway, as witnessed by a near two-fold increase of the relative acetyl-CoA production *via* the synthetic route. Finally, optimizing the expression of the MVA pathway genes increased MVA production to 2.9 mM after 24 h, which is in the range of other studies investigating optimized MVA production from glucose (Zhang et al., 2024). However, the contribution of the SAGA pathway to acetyl-CoA production remained comparatively low and did not exceed 9.3% even in the most advanced strain. Evidently the synthetic pathway is still not the dominant route for acetyl-CoA production and overcoming the highlighted limitations represents a major challenge for carbon-efficient synthesis from EG. Glycolate and oxalate remained major by-products, suggesting that elimination of unidentified GA-dehydrogenases and/or optimization of the GA-consuming Ara5P aldolase enzyme will be required to achieve higher carbon-flux through the SAGA pathway. Potential strategies to identify unknown GA-dehydrogenases include conducting sequence similarity searches for *E. coli*’s known GA dehydrogenase AldA against the proteome of *P. putida* KT2440, or transcriptome profiling of GA-responsive genes.

In summary, by (I) exploiting the thermodynamically favorable PQQ-dependent EG oxidation of *P. putida*, (II) removing competing native GA-dehydrogenases (III) relieving cofactor competition through *gcd* deletion, (IV) supplying glucose to regenerate GA3P and (V) optimizing GA3P availability, we gradually shifted EG-derived carbon flux towards the engineered SAGA pathway, laying the groundwork for further optimization of acetyl-CoA production from EG.

## Data Availability

The original contributions presented in the study are included in the article/Supplementary Material, further inquiries can be directed to the corresponding author.
