# Supplementary material for: Implementation of the synthetic arabinose 5-phosphate dependent glycolaldehyde assimilation (SAGA) pathway in Pseudomonas putida
Source: Front Bioeng Biotechnol. 2026 Aug 25;14:1875703. doi: 10.3389/fbioe.2026.1875703 (PMC13550131; doi:10.3389/fbioe.2026.1875703)
Supplement: Supplementary file 2 [file Supplementaryfile2.docx]

Supplementary Material

Implementation of the synthetic Arabinose 5-phosphate dependent glycolaldehyde assimilation (SAGA) pathway in *Pseudomonas putida*

Alrik Titze^1^, Nils Wagner^1^, Cláudio Frazão^1^_,_ Thomas Walther^1^

^1^Chair of bioprocess engineering, Institute of Natural Materials Technology, TU Dresden, Dresden, Germany

*** Correspondence:**
Thomas Walther
thomas_walther@tu-dresden.de

# Contents Summary:

Material and Methods

Table S1: Plasmids used in this study including primers used for construction

Table S2: Primers used in this study

Table S3: Alignment results from the P. putida KT2440 genome against the known GA reductases Ec.YqhD, Ec.FucO and Go.Gox0313

Table S4: Growth rates of SMP7 and SMP9 on glucose

Figure S1: SAGA pathway enzymatic activity for initial production strain SMP1 (*P. putida* KT2440–Ec.atoB-Lc.mvaS-Lc.mvaE-Ec.KdsD(c-opt)-Ec.Rpe-Ec.Pta + pBTL2-Ec.FsaA(c-opt)-Ca.Pkt(c-opt))

Figure S2: Glycolate production and ^13^C labeling data of SMP1 (*P. putida* KT2440–Ec.atoB-Lc.mvaS-Lc.mvaE-Ec.KdsD(c-opt)-Ec.Rpe-Ec.Pta + pBTL2-Ec.FsaA(c-opt)-Ca.Pkt(c-opt)) after 24 h, depending on initial cell density and presence of glucose.

Figure S3: Substrate consumption and extracellular product accumulation for (A) SMP4, (B) SMP5, (C) SMP6 and (D) SMP7.

Figure S4: OD_600_ and Glucose consumption of SMP7, SMP8 and SMP9.

References

# Material and Methods

Construction of pNPTS138-R6KT derived suicide vectors

Plasmids for gene deletions except pNPTS138-R6KT-dglcDEF and pNPTS138-R6KT-dgapA were constructed by PCR amplifying 500-1000 base pair sequences upstream and downstream of the deletion target gene from genomic *P. putida* KT2440 DNA. The forward primer of the upstream sequence and the reverse primer of the downstream sequence introduced target sites for specific restriction enzymes (listed in Table S2). As indicated in Table S2, upstream and downstream sequences were assembled by either adding restriction sites as described above or splicing by overhang extension using primers containing overhangs complementary to the adjacent sequence. The insert consisting of up- and downstream sequences was then digested with the indicated restriction enzymes and ligated to the pNPTS138-R6KT backbone which had been digested with corresponding restriction enzymes, using T4 DNA ligase (New England Biolabs). Plasmids pNTPS138-R6KT-dglcDEF and pNPTS138-R6KT-dgapA were constructed by amplifying 500-1000 base pair sequences upstream and downstream of the deletion target gene with primers containing 10-20 base pair tails homologous to the adjacent fragment (Table S2). The pNPTS138-R6KT backbone was linearized using PCR. Both DNA sequences and the backbone were subsequently joined via DNA assembly using the NEBuilder HiFi DNA Assembly kit (New England Biolabs).
To construct plasmids for gene integrations, first the base plasmids only containing homologous regions flanking the specific integration site, the constitutive promoter J23100 (Sanches-Medeiros, Monteiro and Silva-Rocha, 2018; Amarelle *et al.*, 2019) as well as flanking terminators (Chaves *et al.*, 2020) were created. For this, the 500-1000 base pair sequences up- and downstream from the integration site were PCR amplified from genomic *P. putida* KT2440 DNA. All primers introduced target sites for indicated restriction enzymes (Table S2). Additionally, the reverse primer of the upstream sequence and the forward primer of the downstream sequence introduced the promoter and terminator sequences. The insert consisting of up- and downstream sequences was then digested with the indicated restriction enzymes and ligated to the digested pNPTS138-R6KT backbone using T4 DNA ligase.	The plasmids pNPTS138-R6KT-PP_5042::J23100-ec.kdsD-ec.rpe and pNPTS138-R6KT-PP_1035::J23100-Ec.atoB-Lc.mvaS-Lc.mvaE were created by PCR amplifying the gene sequences including RBS (Table S1) from previously created plasmids with primers introducing restriction sites for the subsequent digestion and ligation into the digested base plasmid as described above. The plasmids pNPTS138-R6KT-PP_5322::J23100-ec.pta and pNPTS136-R6KT-PP_5042::J23100-ec.kdsD(c-opt)-ec.rpe were created by amplifying the *pta* sequence from *E.coli* MG1655 genomic DNA and the codon optimized *kdsD* synthetic gene using primers containing 10-20 base pair tails homologous to the adjacent sequences in the final plasmid. The *pta* sequence was then integrated into the linearized bas plasmid pNPTS138-R6KT-PP_5322::J23100 and the codon optimized *kdsD* sequence was integrated replacing the *kdsD* sequence in the linearized base-plasmid pNPTS138-R6KT-PP_5042::J23100-ec.kdsD-ec.rpe using DNA assembly.

Construction of pBTL2 gene expression plasmids

All pBTL2 plasmids were created by amplifying codon optimized variants of the *fsaA* from *E.coli* K-12 MG1655 and the *Pkt* from *Clostridium acetobutylicum* ATCC824 from synthetic genes and/or MVA pathway genes (*atoB* from E. coli K-12 MG1655 and *mvaS* as well as *mvaE* from *Lactobacillus casei*) from the pMEV-7 vector (Xiong *et al.*, 2014). Primers contained 15-20 bp tails homologous to sequences adjacent in the final plasmid. Subsequently gene sequences were integrated into the PCR-linearized pBTL2 backbone by *in-vitro* DNA assembly.

Measurement of in-vitro enzyme activities

Phosphoketolase (Pkt) activity was determined using a colorimetric ferric-acetyl-hydroxamate assay described by Racker, 1962. First, 60 µL of test solution containing reaction buffer (53.3 mM HEPES; 26.7 mM KH_2_PO_4_; pH7), 23 mM sodium fluoride, 5 mM magnesium chloride, 1.9 mM l-cysteine, 8 mM iodoacetate, 1 mM thiamine pyrophosphate and 33.3 mM F6P (substrate) were added to 20 µL of cell free extract dilutions and incubated at 30 °C for 30 min. The reaction was then stopped by adding 60 µL of 2 M hydroxylamine chloride solution. Following an incubation time of 10 min at room temperature, 120 µL of an equal mix of 5% iron(III)-chloride in 0.1 M HCl, 15% (v/v) trichloroacetic acid and 4 M HCl were added, causing the ferric-acetyl-hydroxamate complex formation, which was measured at 505 nm. Background signal was determined using test solution without F6P, following the explained procedure. The assay was calibrated by adding test solution without F6P to lithium acetyl phosphate standards instead of cell free extract.

Fructose 6-phosphate aldolase (FsaA) activity was measured on F6P using a protocol adapted from Schürmann and Sprenger, 2001 utilizing the two commercial (Sigma-Aldrich, USA) helper enzymes triose phosphate isomerase (TPI) and glycerol 3-phosphate dehydrogenase (GAPDH) isolated from rabbit muscle. FsaA converts F6P to dihydroxyacetone (DHA) and glyceraldehyde 3-phosphate (GA3P), TPI then converts GA3P to dihydroxyacetone phosphate (DHAP). This is followed by the reduction of DHAP to glycerol 3-phosphate (G3P) by GAPDH which requires the oxidation of NADH to NAD. Decrease of NADH concentration was quantified at 340 nm (ε_340 nm_ = 6.22 mM cm^-1^). For this assay 100 µL of a 62.5 mM F6P solution and 125 µL of test solution containing 100 mM HEPES buffer (pH 7), 1 mM NADH, 80 U mL^-1^ GPDH and 80 U mL^-1^ TPI were added to 25 µL of cell free extract dilutions. The mix was incubated in 96-well plates for 20 minutes at 30 °C while measuring absorption at 340 nm every 20 seconds. Background signal was determined by replacing the F6P solution with water.

Phosphate acetyltransferase (Pta) activity was measured according to (Campos‐Bermudez *et al.*, 2010) by quantifying the thioester bond at 233 nm (ε_233_ _nm_ = 5.55 mM^-1^ cm^-1^) which is formed when supplied acetyl-phosphate is converted to acetyl-CoA. In UV-translucent 96-well plates, 200 µL of a test solution containing 50 mM TRIS/HCl (pH 8.0), 20 mM KCl, 10 mM lithium acetyl phosphate, 0.2 mM disodium CoA and 2 mM dithiothreitol were added to 50 µL of cell free extract dilutions. The mix was incubated for 15 minutes at 30 °C while measuring absorption at 340 nm every 20 seconds Background signal was determined by replacing the lithium acetyl phosphate with water.

**Table S1: Plasmids used in this work including primers used for construction**

| **Name** | **Relevant characteristics** | **Primers used for construction** | **References/origin** |
| --- | --- | --- | --- |
| **Base plasmids** | | | |
| pNPTS138-R6KT | *mobRP4+*; R6K ori; *sacB*; *kan^R^;* suicide plasmid for in-frame deletions |  | (Lassak *et al.*, 2010) |
| pBTL2 | BBR1 ori; P_lac_ promoter, *kan^R^* |  | (Lynch and Gill, 2006) |
| p-MEV7 | *colE1* ori; P_LlacO_ promoter, Amp^R^, *lacI^q^* carrying *atoB* from E.coli as well as *mvaS* and *mvaE* from *L. casei* |  | (Xiong *et al.*, 2014) |
| pNPTS138-R6KT-PP_1035::J23100 | pNPTS138-R6KT carrying Promoter J23100, flanking terminators from (Chaves *et al.*, 2020), flanked by homologous sequences upstream and downstream of intergenic region between PP_1035 and PP_1036 | TW1097, TW1098, TW1099, TW1100 | This study |
| pNPTS138-R6KT-PP_5322::J23100 | pNPTS138-R6KT carrying Promoter J23100 and flanking terminators from (Chaves *et al.*, 2020), flanked by homologous sequences upstream and downstream of intergenic region between PP_5322 and PP_5323 | TW1105, TW1106, TW1107, TW1108 | This study |
| pNPTS138-R6KT-PP_5042::J23100 | pNPTS138-R6KT carrying Promoter J23100 and flanking terminators from (Chaves *et al.*, 2020), flanked by homologous regions upstream and downstream of intergenic region between PP_5042 and PP_5043 | TW1101, TW1102, TW1103, TW1104 | This study |
| pNPTS138-R6KT-PP_5042::J23100-ec.kdsD-ec.rpe | pNPTS138-R6KT-PP_5042::J23100 carrying *kdsD* from *E.coli* directly following RBS sequence GAATTCATTAAAGAGGAGAAAGGTACC and *rpe* from *E.coli* directly following RBS sequence TCTACGCCTGCGAATATTAGAAATATATTTACTAAGGAGGTTTTTT [base plasmid used to create kdsD and rpe integration plasmid below by exchanging kdsD with codon optimized variant] | TW1112, TW1113 | This study |
| **Plasmids for genomic integration** | | | |
| pNPTS138-R6KT-PP_1035::J23100-Ec.atoB-Lc.mvaS-Lc.mvaE | pNPTS138-R6KT-PP_1035::J23100 carrying *atoB* from *E.coli, mvaS* and *mvaE* from *L. casei* amplified together from pMEV-7 | TW1110, TW 1111 | This study |
| pNPTS138-R6KT-PP_5322::J23100-ec.pta | pNPTS138-R6KT-PP_5322::J23100 carrying *pta* from *E.coli*, directly following RBS sequence GTTTAACTTTAAGAAGGAGATATACC | TW2529, TW2530, TW2370, TW2371, | This study |
| pNPTS138-R6KT-PP_5042::J23100-ec.kdsD(c-opt)-ec.rpe | pNPTS138-R6KT-PP_5042::J23100 carrying *kdsD* (codon optimized) from *E.coli* directly following RBS sequence GAATTCATTAAAGAGGAGAAAGGTACC and *rpe* from *E.coli* directly following RBS sequence TCTACGCCTGCGAATATTAGAAATATATTTACTAAGGAGGTTTTTT | TW2374, TW2375, TW2376, TW2377 | This study |
| **Plasmids for gene deletions** | | | |
| pNPTS138-R6KT-daldB-I | pNPTS138-R6KT carrying homologous sequences upstream and downstream of *aldB-I* (PP_0545) | KS001n, KS002n, KS003, KS004 | This work |
| pNPTS138-R6KT-daldB-II | pNPTS138-R6KT carrying homologous sequences upstream and downstream of *aldB-II* (PP_2680) | KS005, KS006, KS007, KS008 | This work |
| pNPTS138-R6KT-dPP_2492 | pNPTS138-R6KT carrying homologous sequences upstream and downstream of *yqhD* (PP_2492) | TW1206, TW1207, TW1208, TW1209 | This work |
| pNPTS138-R6KT-dPP_2650 | pNPTS138-R6KT carrying homologous sequences upstream and downstream of *gbd* (PP_2650) | TW1202, TW1203, TW1204, TW1205 | This work |
| pNPTS138-R6KT-dPP_2682 | pNPTS138-R6KT carrying homologous sequences upstream and downstream of *yiaY* (PP_2682) | TW1190. TW1191, TW1192, TW1193 | This work |
| pNPTS138-R6KT-dPP_2803 | pNPTS138-R6KT carrying homologous sequences upstream and downstream of *dhaT* (PP_2803) | TW1194, TW1195, TW1196, TW1197 | This work |
| pNPTS138-R6KT-dPP_2049 | pNPTS138-R6KT carrying homologous sequences upstream and downstream of PP_2049 | TW1198, TW1199, TW1200, TW1201 | This work |
| pNPTS138-R6KT-dPP_3839 | pNPTS138-R6KT carrying homologous sequences upstream and downstream of *adhP* (PP_3839) | TW1210, TW1211, TW1212, TW1213 | This work |
| pNPTS138-R6KT-dPP_2426 | pNPTS138-R6KT carrying homologous sequences upstream and downstream of *calA* (PP_2426) | TW1421, TW1422, TW1423, TW1424 | This work |
| pNPTS138-R6KT-dgcd | pNPTS138-R6KT carrying homologous sequences upstream and downstream of *gcd* (PP_1444) | TW1721, TW1722, TW1723, TW1724 | This work |
| pNPTS138-R6KT-dglcDEF | pNPTS138-R6KT carrying homologous sequences upstream and downstream of *glcDEF* (PP_3745-3747) | TW2902, TW2903, TW2904, TW2905, TW2482, TW2483 | This work |
| pNPTS138-R6KT-dgapA | pNPTS138-R6KT carrying homologous sequences upstream and downstream of *gapA* (PP_1009) | TW2619, TW2620, TW2621, TW2622, TW2482, TW2483 | This work |
| **Plasmids for gene expression** | | | |
| pBTL2_ec.fsaA(c-opt)-ca.pkt(c-opt) | pBTL2 carrying codon optimized *fsaA* from *E. coli* following RBS sequence CACGAACTCGGCGGTTACGCCAATTAAATAAGGAGGTTTTTT and codon optimized *pkt* from *C. acetobutylicum* following RBS sequence CTGATCGACCCCAGATAATCACAAGAGGAATAGTAAGGAGGTTTTTA | TW2382, TW2383, TW2378, TW2379, TW2380, TW2381 | This work |
| pBTL2_ec.fsaA(c-opt) | pBTL2 carrying codon optimized *fsaA* from *E.coli* (RBS same as above) | TW2383, TW2500, TW2378, TW2618 | This work |
| pBTL2_ca.ptk(c-opt) | pBTL2 carrying codon optimized *pkt* from *C. acetobutylicum* (RBS same as above) | TW2367, TW2500, TW2368, TW2369 | This work |
| pBTL2_ec.fsaA(c-opt)-ca.pkt(c-opt)-Ec.atoB-Lc.mvaS-Lc-mvaE | pBTL2 carrying *atoB* and codon optimized *fsaA* from *E. coli,* codon optimized *pkt* from *C. acetobutylicum,* as well as *mvaS* and *mvaE* from *L. casei* | TW3638, TW2783, TW3639, TW2500 | This work |

**Table S2: Primers used in this work**
* Restriction sites are cursive, primer overhangs are underlined and gene start/end codons are bold, ** SOE: splicing by overhang extension

| **Name** | **Sequence*** |  | |  |
| --- | --- | --- | --- | --- |
| **Primers for PCR-restriction cloning of integration plasmids** | | **Restriction enzmye** | **Application** | |
| TW1097 | TAAGCA*ACTAGT*ACTTGCATGGCATGAGCTTCA | SpeI | Amplification of homology sequence upstream of **PP_1035** integration site (fw) | |
| TW1098 | TGCTTA*CTCGAG*GCTAGCACTGTACCTAGGACTGAGCTAGCCGTCAAAAGGTTGAAAAATAAAAACGGCGCTAAAAAGCGCCGTTTTTTTTGACGGTGGTACCTGTAGGAGCGGGCTTG | XhoI | Amplification of homology sequence upstream of **PP_1035** integration site (rv) | |
| TW1099 | TAAGCA*CTCGAG*TAGGGATAACAGGGTAATAGACGAACAATAAGGCCTCCCTAACGGGGGGCCTTTTTTATTGATAACAAAATTACATCGGCGACGACTTATC | XhoI | Amplification of homology sequence downstream of **PP_1035** integration site (fw) | |
| TW1100 | TGCTTA*GGATCC*TCGACGTACTTGGCTATGTCG | BamHI | Amplification of homology sequence downstream of **PP_1035** integration site (rv) | |
| TW1101 | TAAGCA*ACTAGT*GGCTTCGAACCAGTCATGGTC | SpeI | Amplification of homology sequence upstream of **PP_5042** integration site (fw) | |
| TW1102 | TGCTTA*CTCGAG*GCTAGCACTGTACCTAGGACTGAGCTAGCCGTCAAAAGGTTGAAAAATAAAAACGGCGCTAAAAAGCGCCGTTTTTTTTGACGGTGGTAGCATCGCCACAGAATCGAGC | XhoI | Amplification of homology sequence upstream of **PP_5042** integration site (rv) | |
| TW1103 | TAAGCA*CTCGAG*TAGGGATAACAGGGTAATAGACGAACAATAAGGCCTCCCTAACGGGGGGCCTTTTTTATTGATAACAAAACGAGCTGGGCATGAAGCAC | XhoI | Amplification of homology sequence downstream of **PP_5042** integration site (fw) | |
| TW1104 | TGCTTA*GGATCC*GTCATCGATGTTGCCGACACT | BamHI | Amplification of homology sequence downstream of **PP_5042** integration site (rv) | |
| TW1105 | TAAGCA*ACTAGT*CACCATGAGCACCACTACAG | SpeI | Amplification of homology sequence upstream of **PP_5322** integration site (fw) | |
| TW1106 | TGCTTA*CTCGAG*GCTAGCACTGTACCTAGGACTGAGCTAGCCGTCAAAAGGTTGAAAAATAAAAACGGCGCTAAAAAGCGCCGTTTTTTTTGACGGTGGTAGGCCATCAATCCAGCTTCAG | XhoI | Amplification of homology sequence upstream of **PP_5322** integration site (rv) | |
| TW1107 | TAAGCA*CTCGAG*TAGGGATAACAGGGTAATAGACGAACAATAAGGCCTCCCTAACGGGGGGCCTTTTTTATTGATAACAAAACAATCGGTCAGGGGTTGCC | XhoI | Amplification of homology sequence downstream of **PP_5322** integration site (fw) | |
| TW1108 | TGCTTA*GGATCC*GGTACTGCTGAACGCGAAAACCT | BamHI | Amplification of homology sequence downstream of **PP_5322** integration site (rv) | |
| TW1110 | TAAGCA*CTCGAG*GAATTCATTAAAGAGGAGAAAGGTACC**ATG**AAAAATTGTGTCATCG | XhoI | Amplification of MVA gene cassette (*Ec.atoB, Lc.mvaS, Lc.mvaE*) from p-MEV7 (fw) | |
| TW1111 | TGCTTA*ATTACCCTGTTATCCCTA***TTA**ATCCCGATTTTCATCTTTTGATTGGCCTACTTTC | SpeI | Amplification of MVA gene cassette (*Ec.atoB, Lc.mvaS, Lc.mvaE*) from p-MEV7 (rv) | |
| TW1112 | TAAGCA*CTCGAG*GAATTCATTAAAGAGGAGAAAGGTACC**ATG**TCGCAC | XhoI | Amplification of kdsD + rpe sequence for integration into R6KT-PP_5042::J23100-ec.kdsD-ec.rpe (fw) | |
| TW1113 | TGCTTA*ATTACCCTGTTATCCCTA***TTA**TTCATGACTTACCTTTGCCAGTTCACTGC | SpeI | Amplification of kdsD + rpe sequence for integration into R6KT-PP_5042::J23100-ec.kdsD-ec.rpe (rv) | |
| **Primers for overhang PCR (DNA assembly) to create integration plasmids** | | **Application** | | |
| TW2370 | ATATACC**ATG**TCCCGTATTATTATGCTGATCCCTA | Amplification of *Ec.pta* gene (fw) | | |
| TW2371 | TATCCCTA**TTA**CTGCTGCTGTGCAGACTGAA | Amplification of *Ec.pta* gene (rv) | | |
| TW2374 | AAAGGTACC**ATG**ACGCATGTAGAACTCCAA | Amplification of *Ec.kdsD* (codon optimized) (fw) | | |
| TW2375 | AGGCGTAGA**TCA**CACCACACCGGCA | Amplification of *Ec.kdsD* (codon optimized) (rv) | | |
| TW2376 | TGGTGTGATCTACGCCTGCGAATATTAGAA | Amplification of R6KT-PP_5042::J23100-ec.rpe backbone including J23100 promoter, kdsD RBS, *Ec.rpe* gene and RBS and flanking terminators (fw) | | |
| TW2377 | TGCGTCATGGTACCTTTCTCCTCTTTAATGAATTC | Amplification of R6KT-PP_5042::J23100-ec.rpe backbone including J23100 promoter, kdsD RBS, *Ec.rpe* gene and RBS and flanking terminators (rv) | | |
| TW2529 | AGCAGTAATAGGGATAACAGGGTAATAGACGAAC | Amplification of R6KT-PP_5322::J23100 backbone including J23100 promoter, RBS, flanking terminators (fw) | | |
| TW2530 | CGGGACATGGTATATCTCCTTCTTAAAGTTAAACCTCG | Amplification of R6KT-PP_5322::J23100 backbone including J23100 promoter, RBS and flanking terminators (rv) | | |
| **Primers for PCR-restriction cloning of deletion plasmids** | | **Restriction enzmye** | **Application** | |
| KS001n | TGCA*ACTAGT*GTGCTCGTTCTGATGGATG | SpeI | Amplification of homology sequence downstream **PP_0545** (fw) | |
| KS002n | TCAG*GCGGCCGC*CATGGTGTGTCTCCTTGG | NotI | Amplification of homology sequence downstream **PP_0545** (rv) | |
| KS003 | ATGCACTAGT*GCGGCCGC*TTCTAGACCGCGTCGCCTTCTT | NotI | Amplification of homology sequence upstream **PP_0545** (fw) | |
| KS004 | ATGC*GCATGC*ACGGCAGGAAGCTGCTGG | SphI | Amplification of homology sequence upstream **PP_0545** (rv) | |
| KS005 | ATGC*GGATCC*ACAAGGACGGCGGCAAGG | BamHI | Amplification of homology sequence upstream **PP_2680** (fw) | |
| KS006 | ATGC*TCTAGA*CATGCTGAGCCTCTGCGG | XbaI | Amplification of homology sequence upstream **PP_2680** (rv) | |
| KS007 | ATGC*ACTAGT*GCGGCCGCCTGGTGAGCTACGACATCAA | SpeI | Amplification of homology sequence downstream **PP_2680** (fw) | |
| KS008 | ATGC*GCATGC*AGTAGTCGATACCCTGGGC | SphI | Amplification of homology sequence downstream **PP_2680** (rv) | |
| TW1190 | TAAGCA*ACTAGT*CTCCGGCACAAGAAGAGGAC | SpeI | Amplification of homology sequence upstream **PP_2682** (fw) | |
| TW1191 | CGGTAAGCCTGTTCTTATTGTTC | (SOE** PCR complement below) | Amplification of homology sequence upstream **PP_2682** (rv) | |
| TW1192 | GAACAATAAGAACAGGCTTACCGGTGCTGGTGGAGCACGTGGTG | (SOE** PCR complement above) | Amplification of homology sequence downstream **PP_2682** (fw) | |
| TW1193 | TGCTTA*GAATTC*AGCAATTCATCGGTACGCGCAGC | EcoRI | Amplification of homology sequence downstream **PP_2682** (rv) | |
| TW1194 | TAAGCA*ACTAGT*CCGGCCTTGCGTGACTAC | SpeI | Amplification of homology sequence upstream **PP_2803** (fw) | |
| TW1195 | TGTCGAAAGGGCAGTGGAG | (SOE** PCR complement below) | Amplification of homology sequence upstream **PP_2803** (rv) | |
| TW1196 | CTCCACTGCCCTTTCGACACGTGATGCCGATATCGTCCGCCATG | (SOE** PCR complement above) | Amplification of homology sequence downstream **PP_2803** (fw) | |
| TW1197 | TGCTTA*GAATTC*ATGATGACCAGTTGCACCACCATCAACAG | EcoRI | Amplification of homology sequence downstream **PP_2803** (rv) | |
| TW1198 | TAAGCA*ACTAGT*GCATACAGGCCCTCGACC | SpeI | Amplification of homology sequence upstream **PP_2049** (fw) | |
| TW1199 | CGCACTCTCCTTGTTCGTGTG | (SOE** PCR complement below) | Amplification of homology sequence upstream **PP_2049** (rv) | |
| TW1200 | CACACGAACAAGGAGAGTGCGGACTACGCCGAATTGGCCGTG | (SOE** PCR complement above) | Amplification of homology sequence downstream **PP_2049** (fw) | |
| TW1201 | TGCTTA*GAATTC*TGGCTTTGCCGATAGGGGTG | EcoRI | Amplification of homology sequence downstream **PP_2049** (rv) | |
| TW1202 | TAAGCA*ACTAGT*CTGCCTTCATCAGCAACG | SpeI | Amplification of homology sequence upstream **PP_2650** (fw) | |
| TW1203 | AAGGCTTGGCAGGCTCCT | (SOE** PCR complement below) | Amplification of homology sequence upstream **PP_2650** (rv) | |
| TW1204 | AGGAGCCTGCCAAGCCTTAATGAACACAAGATGCAACGCATC | (SOE** PCR complement above) | Amplification of homology sequence downstream **PP_2650** (fw) | |
| TW1205 | TGCTTA*GAATTC*GCTTCTCGGCCTCGAACC | EcoRI | Amplification of homology sequence downstream **PP_2650** (rv) | |
| TW1206 | TAAGCA*ACTAGT*ACTGCTCTGGAGCACAAC | SpeI | Amplification of homology sequence upstream **PP_2492** (fw) | |
| TW1207 | AGATTTCTCTACTCCTTAAAAGTAGACCAG | (SOE** PCR complement below) | Amplification of homology sequence upstream **PP_2492** (rv) | |
| TW1208 | CTGGTCTACTTTTAAGGAGTAGAGAAATCTCCTATCGGCCTACCAGCT | (SOE** PCR complement above) | Amplification of homology sequence downstream **PP_2492** (fw) | |
| TW1209 | TGCTTA*GAATTC*GATGGAGCGCTCAATCAAGG | EcoRI | Amplification of homology sequence downstream **PP_2492** (rv) | |
| TW1210 | TAAGCA*ACTAGT*TAGAGAAATTCGTGGTCAAGCAGGTG | SpeI | Amplification of homology sequence upstream **PP_3839** (fw) | |
| TW1211 | GAGAGCCTCCGTGTCTATCC | (SOE** PCR complement below) | Amplification of homology sequence upstream **PP_3839** (rv) | |
| TW1212 | GGATAGACACGGAGGCTCTCAAAGTGGTGCCCAAGGTAAC | (SOE** PCR complement above) | Amplification of homology sequence downstream **PP_3839** (fw) | |
| TW1213 | TGCTTA*GAATTC*GTCTTCAGCATGAAGGATGAGTG | EcoRI | Amplification of homology sequence downstream **PP_3839** (rv) | |
| TW1421 | TAAGCA*ACTAGT*CTTCGCCAATCAGCATCTG | SpeI | Amplification of homology sequence upstream **PP_2426** (fw) | |
| TW1422 | CTCGCAGTGAAGTGAAACGATTC | (SOE** PCR complement below) | Amplification of homology sequence upstream **PP_2426** (rv) | |
| TW1423 | GAATCGTTTCACTTCACTGCGAGTTGATCGGTTGCCTGTAC | (SOE** PCR complement above) | Amplification of homology sequence downstream **PP_2426** (fw) | |
| TW1424 | TGCTTA*GAATTC*GGTATCTTCGTACTGGCCAC | EcoRI | Amplification of homology sequence downstream **PP_2426** (rv) | |
| TW1721 | TAAGCA*ACTAGT*TCTCAAGGACAGCAACGGTC | SpeI | Amplification of homology sequence upstream **PP_1444** (fw) | |
| TW1722 | CGTAGGTTCTCCGTCAGGTC | (SOE** PCR complement below) | Amplification of homology sequence upstream **PP_1444** (rv) | |
| TW1723 | GACCTGACGGAGAACCTACGGCTTACAAATTAGCCGAGTAAGC | (SOE** PCR complement above) | Amplification of homology sequence downstream **PP_1444** (fw) | |
| TW1724 | TGCTTA*GAATTC*TGCCGAGGTGTCGAAGTG | EcoRI | Amplification of homology sequence downstream **PP_1444** (rv) | |
| **Primers for overhang PCR (DNA assembly) to create integration plasmids** | | **Application** | | |
| TW2483 | GAATTCGCTAGCTTCGGCC | Amplification of pNPTS138-R6KT backbone (fw) | | |
| TW2483 | ACTAGTGAGTCGTATTACGTAGCTTG | Amplification of pNPTS138-R6KT backbone (rv) | | |
| TW2619 | GTAATACGACTCACTAGTCGCTCAGCCAGGCTTCAAAGGC | Amplification of homology sequence upstream **PP_1009** (fw) | | |
| TW2620 | GTACTGCCCGCGACCCGTGTGGGAGC | Amplification of homology sequence upstream **PP_1009** (rv) | | |
| TW2621 | GGGTCGCGGGCAGTACCTCTGGCAGTTCG | Amplification of homology sequence downstream **PP_1009** (fw) | | |
| TW2622 | CGAAGCTAGCGAATTCACGAGGCGCCCGGCAG | Amplification of homology sequence downstream **PP_1009** (rv) | | |
| TW2902 | ACGTAATACGACTCACTAGTCAGCAGGTCGAACAGAGTGC | Amplification of homology sequence upstream **glcDEF** (fw) | | |
| TW2903 | CATCGCGCAGCATCGACGCGTTCGTCGTAC | Amplification of homology sequence upstream **glcDEF** (rv) | | |
| TW2904 | CGCGTCGATGCTGCGCGATGAGCCGATCG | Amplification of homology sequence downstream **glcDEF** (fw) | | |
| TW2905 | CGGCCGAAGCTAGCGAATTCCAGCGCGATGTAGGCACTGG | Amplification of homology sequence downstream **glcDEF** (rv) | | |
| **Primers for overhang PCR (DNA assembly) to create gene expression plasmids** | | **Application** | | |
| TW2367 | TTTACCAATAATCGACTG**CAT**TAAAAACCTCCTTACTATTCCTCTTGTGA | Amplification of pBTL2 backbone + P_lac_ + *Ca.pkt* RBS (rv) | | |
| TW2368 | AGTCGATTATTGGTAAACACAAGGATGAGGGCAAGATCACCCCAG | Amplification of *Ca.pkt* (codon optimized) (fw) | | |
| TW2369 | TTATCCCTA**TCA**CACGTGCCACTGC | Amplification of *Ca.pkt* (codon optimized) (rv) | | |
| TW2378 | TAAATAAGGAGGTTTTTT**ATG**GAATTGTACTTGGATACTAG | Amplification of *Ec.fsaA* (codon optimized) (fw) | | |
| TW2379 | CTTGTGATTATCTGGGGTCGATCAG**TCA**GATGCTGGTGCGA | Amplification of *Ec.fsaA* (codon optimized) (rv) | | |
| TW2380 | GCATCTGACTGATCGACCCCAGATAATC | Amplification of *Ca.pkt* (codon optimized) + RBS (fw) | | |
| TW2381 | TATCCCTA**TCA**CACGTGCCACTGC | Amplification of *Ca.pkt* (codon optimized) + RBS (rv) | | |
| TW2382 | CACGTG**TGA**TAGGGATAACAGGGTAATGAT | Amplification of pBTL2 backbone + P_lac_ + *fsaA* RBS (fw) | | |
| TW2383 | AAAAAACCTCCTTATTTAATTGGCG | Amplification of pBTL2 backbone + P_lac_ + *fsaA* RBS (rv) | | |
| TW2500 | TAGGGATAACAGGGTAATGATATC | Amplification of pBTL2 backbone + P_lac_ + *fsaA* RBS (fw) | | |
| TW2618 | ATTACCCTGTTATCCCTA**TCA**GATGCTGGTGCGACCGA | Amplification of *Ec.fsaA* (codon optimized) (rv) | | |
| TW2783 | ATTACCCTGTTATCCCTA**TTA**ATCCCGATTTTCATCTTTTGATTG | Amplification of *Ec.atoB, Lc.mvaS, Lc.mvaE* + all 3 RBS (rv) | | |
| TW3638 | CACGTG**TGA**CACGAACTCGGCGGTTAC | Amplification of *Ec.atoB, Lc.mvaS, Lc.mvaE* + all 3 RBS (fw) | | |
| TW3639 | GTTCGTG**TCA**CACGTGCCACTGC | Amplification of pBTL2 backbone including *Ec.fsaA*(c-opt) and *Ca.pkt*(c-opt) + both RBS + P_lac_ (rv) | | |

**Table S3: Alignment results from the P. putida KT2440 genome against the known GA reductases Ec.YqhD, Ec.FucO and Go.Gox0313**
Annotations listed in the table were obtained from the Pseudomonas Genome Database (Winsor *et al.*, 2016)

**Table S4: Growth rates of SMP7 and SMP9 on glucose**
SMP7 and SMP9 were grown from an initial OD600 of 0.2 in M9 + 111 mM glucose. n=3

| **Strain** | **Growth rate µ [h^-1^]** |
| --- | --- |
| SMP7 | 0.27 ± 0.01 |
| SMP9 | 0.14 ± 0.01 |

**Figure S1: SAGA pathway enzymatic activity for initial production strain SMP1 (*P. putida* KT2440–Ec.atoB-Lc.mvaS-Lc.mvaE-Ec.KdsD(c-opt)-Ec.Rpe-Ec.Pta + pBTL2-Ec.FsaA(c-opt)-Ca.Pkt(c-opt)).** Activities were determined in cell-free extracts of *P. putida* KT2440 wildtype and strain SMP1. Error bars indicate standard error of the mean (n = 3).

**Figure S2:** **Glycolate production and ^13^C labeling data of SMP1 (*P. putida* KT2440–Ec.atoB-Lc.mvaS-Lc.mvaE-Ec.KdsD(c-opt)-Ec.Rpe-Ec.Pta + pBTL2-Ec.FsaA(c-opt)-Ca.Pkt(c-opt)) after 24 h, depending on initial cell density and presence of glucose.**
Cells were inoculated at high cell density (HCD, OD_600_ ~12) or low cell density (LCD, OD_600_ = 0.2) and cultivated in M9 + 50 mM ^13^C_2_-EG supplemented with either 111 mM glucose or glucose-releasing beads. **(A)** Glycolate production during cultivation. **(B)** ^13^C enrichment in mevalonate after 24 h of cultivation. **(C)** Relative isotopologue distribution in acetyl-CoA after 24 h inferred from the corresponding distribution in mevalonate. Error bars indicate the standard error of the mean (n = 3).

**Figure S3:** **Substrate consumption and extracellular product accumulation for (A) SMP4, (B) SMP5, (C) SMP6 and (D) SMP7.**
All strains were inoculated to an OD_600_~12 and cultivated in M9 medium containing 111 mM glucose and 50 mM ^13^C_2_-EG. Error bars indicate standard error of the mean (n = 2).

**Figure S4:** **OD_600_ and Glucose consumption of SMP7, SMP8 and SMP9.**
Strains were inoculated to OD_600_ ≈ 9 and cultivated in M9 medium supplemented with 111 mM glucose and 50 mM ^13^C_2_-EG at 30°C. **(A)** OD_600_ and **(B)** glucose concentration of strains SMP7 (*P. putida* KT2440 *∆aldB-I ∆aldB-II ∆gcd*-Ec.kdsD-Ec.rpe-Ec.pta + pBTL2-Ca.pkt-Ec.fsaA), SMP8 (SMP7 *∆glcDEF*) and SMP9 (SMP7 *∆gapA*). Error bars indicate standard error of the mean (n = 2).

# References

Amarelle, V. *et al.* (2019) “Expanding the Toolbox of Broad Host-Range Transcriptional Terminators for Proteobacteria through Metagenomics,” *ACS Synthetic Biology*, 8(4), pp. 647–654. Available at: https://doi.org/10.1021/acssynbio.8b00507.

Chaves, J.E. *et al.* (2020) “Evaluation of chromosomal insertion loci in the Pseudomonas putida KT2440 genome for predictable biosystems design,” *Metabolic Engineering Communications*, 11, p. e00139. Available at: https://doi.org/10.1016/j.mec.2020.e00139.

Lassak, J. *et al.* (2010) “ArcS, the Cognate Sensor Kinase in an Atypical Arc System of *Shewanella oneidensis* MR-1,” *Applied and Environmental Microbiology*, 76(10), pp. 3263–3274. Available at: https://doi.org/10.1128/AEM.00512-10.

Lynch, M.D. and Gill, R.T. (2006) “Broad host range vectors for stable genomic library construction,” *Biotechnology and Bioengineering*, 94(1), pp. 151–158. Available at: https://doi.org/10.1002/bit.20836.

Sanches-Medeiros, A., Monteiro, L.M.O. and Silva-Rocha, R. (2018) “Calibrating Transcriptional Activity Using Constitutive Synthetic Promoters in Mutants for Global Regulators in *Escherichia coli*,” *International Journal of Genomics*, 2018, pp. 1–10. Available at: https://doi.org/10.1155/2018/9235605.

Winsor, G.L. *et al.* (2016) “Enhanced annotations and features for comparing thousands of *Pseudomonas* genomes in the Pseudomonas genome database,” *Nucleic Acids Research*, 44(D1), pp. D646–D653. Available at: https://doi.org/10.1093/nar/gkv1227.

Xiong, M. *et al.* (2014) “Scalable production of mechanically tunable block polymers from sugar,” *Proceedings of the National Academy of Sciences*, 111(23), pp. 8357–8362. Available at: https://doi.org/10.1073/pnas.1404596111.
